# Wnt3a‐Loaded Extracellular Vesicles Promote Alveolar Epithelial Regeneration after Lung Injury

**DOI:** 10.1002/advs.202206606

**Published:** 2023-04-18

**Authors:** Lei Gao, Yongping Sun, Xinye Zhang, Ding Ma, An Xie, Enyu Wang, Linzhao Cheng, Senquan Liu

**Affiliations:** ^1^ Department of Hematology The First Affiliated Hospital of USTC Division of Life Sciences and Medicine University of Science and Technology of China Hefei Anhui 230027 China; ^2^ Blood and Cell Therapy Institute Anhui Provincial Key Laboratory of Blood Research and Applications University of Science and Technology of China Hefei Anhui 230027 China; ^3^ School of Basic Medical Sciences Division of Life Sciences and Medicine University of Science and Technology of China Hefei Anhui 230027 China

**Keywords:** chronic obstructive pulmonary disease, extracellular vesicles, regeneration, Wnt3a

## Abstract

Compromised regeneration resulting from the deactivation of Wnt/*β*‐catenin signaling contributes to the progression of chronic obstructive pulmonary disease (COPD) with limited therapeutic options. Extracellular cytokine‐induced Wnt‐based signaling provides an alternative option for COPD treatment. However, the hydrophobic nature of Wnt proteins limits their purification and use. This study devises a strategy to deliver the membrane‐bound wingless‐type MMTV integration site family, member 3A (Wnt3a) over a long distance by anchoring it to the surface of extracellular vesicles (EVs). The newly engineered Wnt3a^WG^ EVs are generated by co‐expressing Wnt3a with two genes encoding the membrane protein, WLS, and an engineered glypican, GPC6^ΔGPI^‐C1C2. The bioactivity of Wnt3a^WG^ EVs is validated using a TOPFlash assay and a mesoderm differentiation model of human pluripotent stem cells. Wnt3a^WG^ EVs activate Wnt signaling and promote cell growth following human alveolar epithelial cell injury. In an elastase‐induced emphysema model, impaired pulmonary function and enlarged airspace are greatly restored by the intravenous delivery of Wnt3a^WG^ EVs. Single‐cell RNA sequencing–based analyses further highlight that Wnt3a^WG^ EV‐activated regenerative programs are responsible for its beneficial effects. These findings suggest that EV‐based Wnt3a delivery represents a novel therapeutic strategy for lung repair and regeneration after injury.

## Graphical abstract

The hydrophobic nature of Wnt ligands limits their purification and applications. A strategy is devised to deliver functional Wnt3a by shielding lipid modification with glypican and anchoring to extracellular vesicles (EVs). These engineered EVs (Wnt3a^WG^ EVs) exert therapeutic functions in a murine emphysema model through reinforcing regenerative programs. EV‐based Wnt signaling activation represents a valuable therapeutic strategy for lung diseases.

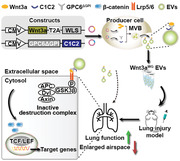

## 1 Introduction

There has been little progress in treatment options for many acute and chronic lung diseases over the past few decades.^[^ ^1^ ^]^ A growing body of evidence demonstrates that endogenous regenerative mechanisms are severely compromised after lung injury.^[^ ^2^ ^]^ Chronic obstructive pulmonary disease (COPD), characterized by persistent airflow obstruction and severe respiratory symptoms, has been a leading cause of disability and death worldwide.^[^ ^2^, ^3^ ^]^ Patients with COPD generally display one of the following two phenotypes: 1) emphysema, characterized by enlargement of distal airspace, or 2) bronchitis, involving inflammation of small airways that results in chronic cough.^[^ ^2a^ ^]^ Bronchodilators and inhaled corticosteroids are the primary pharmacological treatments. While symptoms and dysfunctional performance can be reduced, structural deterioration is inevitable.^[^ ^2^, ^4^ ^]^ Lung volume reduction surgery is an alternative option for the treatment of severe emphysema; however, perioperative surgical complications limit its application.^[^ ^4^ ^]^ Thus, there is an urgent need for novel and effective COPD treatment strategies.

Impaired regeneration resulting from suppressed Wnt signaling contributes to the development of COPD.^[^ ^2^, ^5^ ^]^ Studies have investigated the use of small molecules such as lithium chloride (LiCl) and CHIR99021 to activate the canonical Wnt pathway by inhibiting glycogen synthase kinase‐3 (GSK3) kinases.^[^ ^6^ ^]^ However, the severe toxicity of these molecules limits their therapeutic use. For example, LiCl, an old drug approved for the treatment of bipolar disorder, frequently leads to neurological toxicity, such as parkinsonism and extrapyramidal symptoms caused by excessive iron accumulation in the brain.^[^ ^7^ ^]^ While a recent study highlighted the therapeutic potential of CHIR99021 in combination with valproic acid, an antiepileptic drug, in patients with stable sensorineural hearing loss, this molecule is not pharmacologically suitable for systematic delivery to tissues and organs, such as the lung, due to its broad‐spectrum kinase inhibitory property.^[^ ^8^ ^]^ Thus, directly activating Wnt signaling to trigger endogenous regenerative mechanisms may provide a better option for the treatment of COPD.

Activation of Wnt signaling relies on the binding of extracellular Wnt proteins to their co‐receptors, Frizzleds (FZDs) and LRP5/6, on the cell surface.^[^ ^9^ ^]^ Secreted Wnt glycoproteins are further modified by the addition of palmitoleate and are highly hydrophobic, which has limited their purification and therapeutic use.^[^ ^10^ ^]^ Recombinant Wnt proteins can be produced in mammalian cells and purified using detergents. However, the specific activity of purified Wnt3a is low and not suitable for in vivo study and use. To address this obstacle, some investigators have designed water‐soluble Wnt surrogates.^[^ ^11^ ^]^ These chimeric proteins function as activators by bringing together FZD and LRP surface transducing proteins and activating downstream Wnt signaling.^[^ ^11^, ^12^ ^]^ Other researchers have tried modifying Wnt protein structure. Using this method, Wnt3a has been protected by glypicans or anchored to the surface of extracellular vesicles (EVs), allowing the protein to travel a long distance and activate target cell signaling.^[^ ^10^, ^13^ ^]^ EVs are nanosized membrane vehicles that are secreted by multiple cell types and function as natural intercellular messengers with less immunoreactivity than synthetic vehicles. Given their unique properties, EVs have emerged as an attractive therapeutic vehicle for the delivery of functional proteins.^[^ ^14^ ^]^ An active form of Wnt3a‐overexpressed EVs have been recently delivered into cartilage where it promotes the repair of osteochondral defects.^[^ ^15^ ^]^ Our studies have also shown that EVs derived from human induced pluripotent stem cells (hiPSCs) alleviate cellular aging,^[^ ^16^ ^]^ and serve as a vehicle with magneto encapsulation to allow MRI‐based imaging.^[^ ^17^ ^]^ Since Wnt signaling activation is an attractive therapeutic approach for treating COPD,^[^ ^6^ ^]^ the current study assessed whether EVs can serve as an effective vehicle to deliver functional Wnt3a protein, and facilitate pulmonary repair and regeneration.

## 2 Results

### 2.1 Design and Generation of Functional Wnt3a‐Loaded EVs

To investigate possible roles of Wnt signaling in COPD, altered gene expression was assessed in the lungs of COPD patients. A publicly available single‐cell RNA sequencing (scRNA‐seq) dataset (GSE173896) consisting of six COPD patients and three healthy volunteers were obtained (Figure S1A,B, Supporting Information). Cells were grouped into epithelial, mesenchymal, endothelial, and immune populations (Figure S1C,D, Supporting Information). The expression of Wnt ligands (Figure S1E, Supporting Information) and downstream target genes (Figure S1F, Supporting Information) were suppressed in the epithelial cells of COPD patients. By employing a human alveolar epithelial cell injury model induced by lipopolysaccharides (LPS),^[^ ^6^, ^18^ ^]^ both CHIR99021 and LiCl were found to moderately restore impaired cell growth (Figure S2, Supporting Information), suggesting that Wnt signaling activation may represent a therapeutic option for lung injury‐related diseases. Wnt‐induced regeneration in alveolar epithelial cells also plays a central role in inhibiting lymphotoxin *β*‐receptor signaling.^[^ ^6c^ ^]^ However, Wnt proteins are palmitoylated, rendering them insoluble. To overcome this, and to allow long‐distance signaling, results from previous studies have encouraged the use of EVs to deliver Wnt3a.^[^ ^13^, ^15^ ^]^

**Figure 1:**
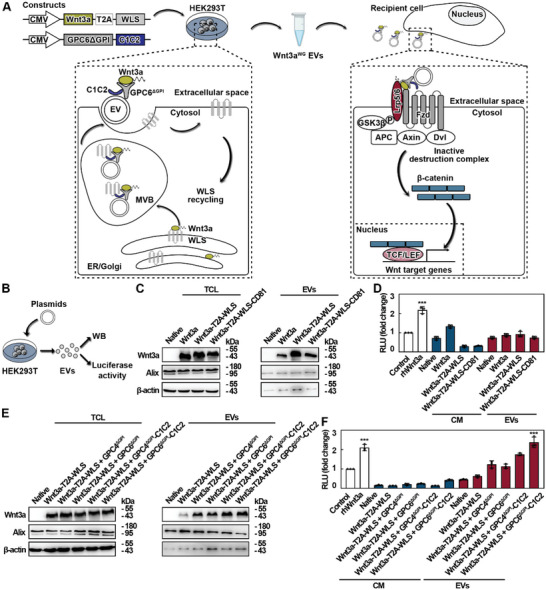
Design and generation of functional Wnt3a‐loaded EVs. A) Schematic illustration of EV‐based strategies to deliver hydrophobic Wnt3a in an active form. B) Schematic of the experimental design for assessment of Wnt3a content and activity. Conditioned medium was generated from HEK293T cells transfected with various plasmids for three consecutive days. EVs were isolated by ultracentrifugation method and then used for western blot (WB) analysis and TOPFlash reporter assay. C) WB analysis of Wnt3a, Alix, and *β*‐actin in EVs and the corresponding donor cells, which were transfected with Wnt3a, Wnt3a‐T2A‐WLS, and Wnt3a‐T2A‐WLS‐CD81 vectors, respectively. Representative images of two independent experiments are shown. TCL, total cell lysate. D) TOPFlash reporter assay in HEK293T cells. Cells transfected with TOPFlash luciferase and pRL‐TK reporter plasmids were treated with different Wnt3a‐loaded EVs and the corresponding conditioned medium (CM) for 48 h; luciferase activity was then detected. RLU, relative luciferase unit. Data are shown as mean ± SD. *, significantly different from control; ***p < 0.001. Two independent experiments were performed. rhWnt3a, recombinant human Wnt3a. E) WB analysis of Wnt3a, Alix, and *β*‐actin in EVs and the corresponding donor cells, which were transfected with Wnt3a‐T2A‐WLS together with GPC4^ΔGPI^, GPC6^ΔGPI^, GPC4^ΔGPI^‐C1C2, and GPC6^ΔGPI^‐C1C2 vectors, respectively. Representative images of three independent experiments are shown. F) TOPFlash reporter assay in HEK293T cells. Cells transfected with TOPFlash luciferase and pRL‐TK reporter plasmids were treated with various Wnt3a‐loaded EVs and the corresponding CM for 48 h; luciferase activity was then detected. Data are shown as mean ± SD. *, significantly different from control; ***p < 0.001. Three independent experiments were performed.

As a proof of concept, Wnt3a‐loaded EVs were generated and their functional activity was assessed (**Figure** **1**A). A simple transfection of one vector expressing Wnt3a was insufficient to observe its biological activity. Additional engineering strategies were employed to load Wnt3a onto EVs in an active form. Wnt3a expression and bioactivity were then determined by Western blot (WB) and TOPFlash assays, respectively (Figure 1B). Since WLS is responsible for the trafficking of Wnt3a from the endoplasmic reticulum to the cell surface and CD81 is highly enriched in EVs,^[^ ^10a^ ^]^ Wnt3a‐T2A‐WLS and Wnt3a‐T2A‐WLS‐CD81 expression vectors were designed and compared to a vector expressing Wnt3a alone. The vectors were successfully designed and constructed (Figure 1C), however, these three types of Wnt3a‐loaded EVs failed to induce strong Wnt activity in the reporter cells transfected with TOPFlash and pRL‐TK plasmids (Figure 1D).

To address this, a membrane anchor was used to offer hydrophobic protection and preserve the bioactivity of EV‐associated Wnt3a.^[^ ^13^, ^19^ ^]^ GPC4^ΔGPI^, GPC6^ΔGPI^, GPC4^ΔGPI^‐C1C2, and GPC6^ΔGPI^‐C1C2 constructs containing an engineered glypican were designed and co‐transfected with the Wnt3a‐T2A‐WLS vector due to its ability to effectively load Wnt3a onto EVs (Figure 1C). Wnt3a overexpression in total cell lysate (TCL) and EVs were also achieved in the presence of glypicans (Figure 1E). Similar to recombinant human Wnt3a (rhWnt3a), Wnt3a‐loaded EVs (Wnt3a^WG^ EVs) obtained from Wnt3a‐T2A‐WLS and GPC6^ΔGPI^‐C1C2 co‐transfected cells led to a significant twofold induction of luciferase activity in the reporter cells (Figure 1F). These findings suggest that biologically active Wnt3a‐loaded EVs (Wnt3a^WG^ EVs) were successfully designed and generated in cells by co‐expressing Wnt3a with WLS (as a secretion booster) and an engineered glypican, GPC6^ΔGPI^‐C1C2 (as a stabilizer anchor).

### 2.2 Characterization of Wnt3a^WG^ EVs

**Figure 2:**
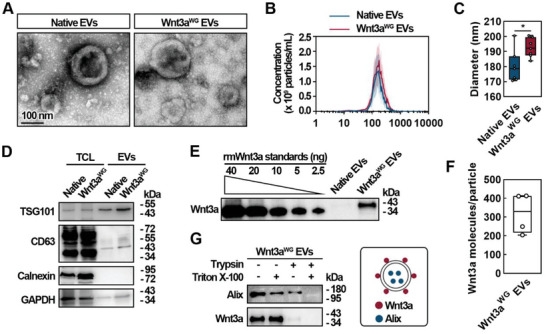
Characterization of Wnt3a^WG^ EVs. A) TEM analysis of Native EVs and Wnt3a^WG^ EVs. Representative images from two biological replicates are shown. B) Size distribution and C) the diameter analyses of Native EVs and Wnt3a^WG^ EVs. Data are shown as mean ± SD from six biological replicates. *, significantly different from Native EVs; *p < 0.05. D) WB analysis of TSG101, CD63, Calnexin, and GAPDH in EVs and the corresponding donor cells, which was transfected with Wnt3a‐T2A‐WLS and GPC6^ΔGPI^‐C1C2. Representative images of three independent experiments are shown. E) WB‐based quantification of Wnt3a in EVs by using a calibration curve of wild type rmWnt3a. Representative image of four biological replicates is shown. F) The amount of Wnt3a per particle calculated from the quantitative results as shown in E. Data are shown as mean ± SD from four biological replicates. G) Wnt3a^WG^ EVs were incubated with or without 0.025% trypsin and 0.1% Triton X‐100 at 37 °C for 30 min; the expression of Wnt3a and Alix, an intra‐vesicular protein, were then detected. Representative images of two independent experiments are shown.

As recommended, a comprehensive characterization of the Wnt3a^WG^ EVs was performed using multiple approaches.^[^ ^20^ ^]^ Transmission electron microscopy (TEM) and nanoparticle tracking analysis (NTA) demonstrated that both Native and Wnt3a^WG^ EVs had a round shape with a 100–200 nm diameter (**Figure** **2**A,B). While the diameter of Wnt3a^WG^ EVs (192.5 ± 5.8 nm) was significantly larger than Native EVs (180.1 ± 9.8 nm) (Figure 2C), the particle concentration and total EV protein levels were comparative (Figure S3, Supporting Information). Immunoblot analysis showed that representative EV markers, including TSG101 and CD63, were enriched in both Native EVs and Wnt3a^WG^ EVs, while an endoplasmic reticulum marker, Calnexin, was absent (Figure 2D), suggesting that isolated EVs were free from cellular contamination. A standard curve derived from recombinant mouse Wnt3a (rmWnt3a) was generated to quantify the amount of Wnt3a loaded onto Wnt3a^WG^ EVs (Figure 2E and Figure S4A, Supporting Information) as previously reported.^[^ ^21^ ^]^ The number of Wnt3a molecules (320 ± 93) loaded onto each EV (Figure 2F) and the amount of Wnt3a protein (Figure S4B, Supporting Information) was calculated using densitometry‐based quantification. To determine the topology of Wnt3a, Wnt3a^WG^ EVs were digested with trypsin in the presence or absence of Triton X‐100.^[^ ^22^ ^]^ While Alix was resistant to trypsin digestion but was degraded following detergent solubilization, Wnt3a was sensitive to trypsin digestion when EVs were intact (Figure 2G). These results demonstrate that Wnt3a is highly enriched on the surface of Wnt3a^WG^ EVs.

### 2.3 Validation of the Bioactivity of Wnt3a^WG^ EVs Using a hiPSC‐Based Mesoderm Differentiation Model

**Figure 3:**
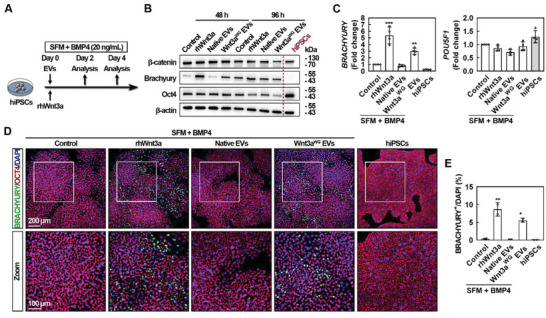
The activity of Wnt3a^WG^ EVs in a hiPSC‐based mesoderm differentiation model. A) Schematic of the experimental design. B) hiPSCs were treated with rhWnt3a (40 ng mL^−1^), Native EVs (2 × 10^9^ particles per mL), and Wnt3a^WG^ EVs (2 × 10^9^ particles per mL) in the presence of BMP4 (20 ng mL^−1^) during mesoderm differentiation induced by serum free medium (SFM) medium for 48 and 96 h, respectively. Cells were then collected for WB analysis. Representative images of three independent experiments are shown. After being treated with rhWnt3a (40 ng mL^−1^), Native EVs (2 × 10^9^ particles per mL), and Wnt3a^WG^ EVs (2 × 10^9^ particles per mL) in the presence of BMP4 (20 ng mL^−1^) during mesoderm differentiation induced by SFM medium for 48 h, cells were collected for C) qRT‐PCR and D,E) immunostaining analyses. For (C), data are shown as mean ± SD from four independent experiments. *, significantly different from Control; **p < 0.01; ***p < 0.001. For (E), data are shown as mean ± SD from two independent experiments. *, significantly different from Control; *p < 0.05; **p < 0.01.

To determine whether the newly engineered EV‐activated Wnt/*β*‐catenin signaling can lead to a phenotypic effect in a biological system, a human pluripotent stem cell (hPSC)‐based mesoderm differentiation model, which is a well‐established cellular platform to study the effect of induced Wnt signal, was thus adopted.^[^ ^12^, ^23^ ^]^ Wnt3a is generally considered as an “amplifier” during the mesodermal fate decision of hPSCs.^[^ ^23^, ^24^ ^]^ Therefore, Wnt3a^WG^ EV and rhWnt3a bioactivity were compared using a hiPSCs‐based mesoderm differentiation model. Initially, a defined culture condition was used to maintain hPSC pluripotency (Figure S5A, Supporting Information). While BRACHYURY protein and mRNA levels were increased after CHIR99021 treatment, they remained unaltered in response to rhWnt3a and Wnt3a^WG^ EV treatment (Figure S5B,C, Supporting Information). In addition, both rhWnt3a and Wnt3a^WG^ EVs were unable to promote BRACHYURY expression under FBS‐based three‐lineage differentiation conditions (Figure S5D–F, Supporting Information).^[^ ^23^ ^]^ The synergy between Wnt3a and BMP4 is reported to facilitate hPSC mesoderm differentiation.^[^ ^24b^ ^]^ Thus, a mesodermal induction medium was used to assess the effect of Wnt3a^WG^ EVs on hiPSC differentiation in the absence or presence of BMP4 (Figure S6A, Supporting Information). Both rhWnt3a and Wnt3a^WG^ EVs promoted BRACHYURY expression in the presence of 10 ng mL^−1^ BMP4 (Figure S6B, Supporting Information). BRACHYURY expression was dramatically increased in response to rhWnt3a and Wnt3a^WG^ EV treatment after a 48 h induction in mesoderm differentiation media supplemented with 20 ng mL^−1^ BMP4, while a 96 h treatment resulted in a robust mesoderm induction (**Figure** **3**A,B). qRT‐PCR and immunostaining were used to confirm these findings. A 48 h treatment with rhWnt3a and Wnt3a^WG^ EVs significantly promoted the expression of BRACHYURY, while the expression of POU5F1 was only slightly altered (Figure 3C). Similar to rhWnt3a (8.6% ± 1.3%), Wnt3a^WG^ EV treatment was able to significantly increase the percentage of BRACHYURY^+^ cells (5.6% ± 0.4%), suggesting that Wnt3a^WG^ EVs and rhWnt3a had comparable mesoderm induction efficiency. These results suggest that the bioactivity of Wnt3a^WG^ EVs and rhWnt3a are comparable in vitro.

### 2.4 Wnt3a^WG^ EVs Protect Human Alveolar Epithelial Cells from LPS‐Induced Injury In Vitro

**Figure 4:**
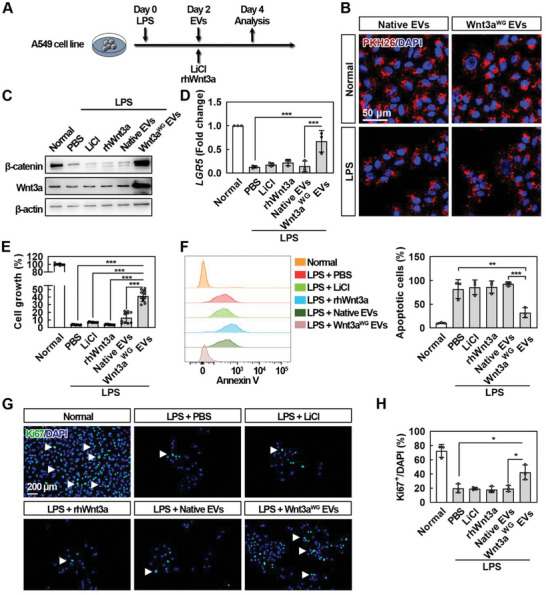
Wnt3a^WG^ EVs promote cell growth, inhibit apoptosis, and facilitate cell proliferation in a human alveolar epithelial cell injury model. A) Schematic of the experimental design. A549 cell line was treated with LPS (50 µg mL^−1^) for 2 days; the treatments including LiCl (5 mm), rhWnt3a (200 ng mL^−1^), Native EVs (2 × 10^9^ particles per mL), and Wnt3a^WG^ EVs (2 × 10^9^ particles per mL) were then added. B) Representative images of cells with 6 h incubation of PKH26‐labeled EVs. Three independent experiments were performed. After being incubated with indicated treatments for 2 days, cells were then collected for C) WB, D) qRT‐PCR, E) CCK‐8, F) Annexin‐V, and G,H) immunostaining analyses, respectively. For (C), representative images of three independent experiments are shown. For (D), three independent experiments were performed. Data are shown as mean ± SD. ***p < 0.001. For (E), data are shown as mean ± SD from two independent experiments. ***p < 0.001. For (F), data are shown as mean ± SD from three independent experiments. **p < 0.01. For (H), data are shown as mean ± SD from three independent experiments. *p < 0.05. Arrows indicate proliferating cells.

The bioactivity of Wnt3a^WG^ EVs was validated using a TOPFlash assay and a hiPSC‐based mesoderm differentiation model. The therapeutic effect of Wnt3a^WG^ EVs was then assessed with an LPS‐induced human alveolar epithelial cell injury model (**Figure** **4**A), using LiCl and rhWnt3a as controls. First, to confirm the cellular uptake in vitro, EVs were labeled with a red fluorescent lipid dye PKH26^[^ ^25^ ^]^ and then incubated with cells for 6 h. As shown in Figure 4B, both PKH26‐labeled Native EVs and Wnt3a^WG^ EVs were internalized by cells in the absence or presence of LPS. Wnt3a^WG^ EVs were also able to deliver Wnt3a into injured alveolar epithelial cells and activate Wnt/*β*‐catenin signaling, as shown by upregulation of *β*‐catenin (Figure 4C), and expression of the downstream target genes, LGR5, AXIN2, and TCF4 (Figure 4D and Figure S7, Supporting Information), which had been suppressed by LPS. It was shown that 2 × 10^9^ particles per mL Wnt3a^WG^ EVs were sufficient to restore LPS‐impaired cell growth, while LiCl, an emphysema drug known to activate Wnt/*β*‐catenin signaling,^[^ ^6b^ ^]^ and rhWnt3a failed to exert this therapeutic effect (Figure 4E and Figure S8, Supporting Information). In addition, Wnt3a^WG^ EVs resulted in a smaller ratio of LPS‐induced apoptotic cells than LiCl and rhWnt3a (Figure 4F). Moreover, Ki67 staining revealed that Wnt3a^WG^ EVs treatment significantly increased the ratio of proliferative cells (Ki67^+^) by more than twofold (Figure 4G,H). Taken together, these findings strengthen the evidence that Wnt signaling prevents apoptosis and maintains cell survival, demonstrating the beneficial effects of Wnt3a^WG^ EVs in vitro.

### 2.5 Wnt3a^WG^ EVs Elicit Alveolar Epithelial Regeneration and Attenuate Lung Injury in a Murine Model of Emphysema

**Figure 5:**
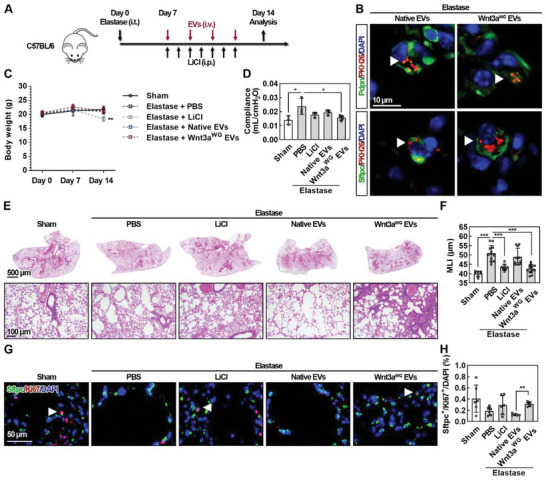
Wnt3a^WG^ EVs restore enlarged airspace in a murine model of emphysema. A) Schematic of the experimental design. Mice were subjected to an intratracheal instillation of elastase (100 U kg^−1^). At day 7, mice were then received intravenous (i.v.) injection of Native EVs (2 × 10^9^ particles per dose) and Wnt3a^WG^ EVs (2 × 10^9^ particles per dose) once every 2 days; intraperitoneal (i.p.) injection of LiCl (200 mg kg^−1^) daily was regarded as a positive control treatment. After treatment for 7 days, mice were sacrificed and subjected for further analyses. B) Representative images of immunofluorescent analysis for Pdpn and Sftpc in lung tissue sections from elastase‐instilled mice after intravenous administration with PKH26‐labeled EVs (red), respectively. Tissue sections from four biological replicates per group were analyzed. C) Body weight analysis of mice. Data are shown as mean ± SD from at least three biological replicates. *, significantly different from sham; **p < 0.01. D) Dynamic compliance analysis of mice receiving various treatments. Data are shown as mean ± SD from at least three biological replicates. *p < 0.05. E) Representative images of H&E‐stained lung tissue sections. F) Quantification of airspace enlargement as mean linear intercepts (MLI). Data are shown as mean ± SD from at least seven biological replicates. ***p < 0.01. Representative images of immunofluorescent analysis for G) Sftpc and Ki67 and quantification of type II alveolar epithelial cells (Sftpc^+^) positive for H) Ki67 in lung tissue sections from mice treated as indicated. Data are shown as mean ± SD from at least five biological replicates. **p < 0.01.

**Figure 6:**
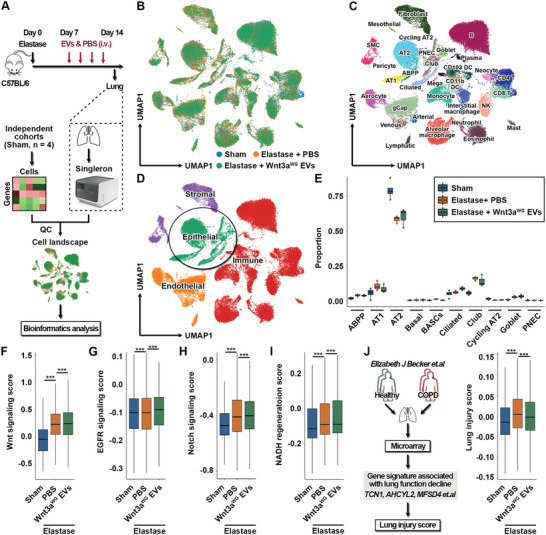
Wnt3a^WG^ EVs reinforce the regenerative and reparative programs in a murine model of emphysema. A) Schematic of the experimental design. Mice were subjected to an intratracheal instillation of elastase (100 U kg^−1^). At Day 7, mice received intravenous injection of Wnt3a^WG^ EVs (2 × 10^9^ particles per dose) and equal volume of PBS once every 2 days. After treatment for 7 days, mice were sacrificed; lung samples were collected for single‐cell RNA sequencing analysis. QC, quality control. B) Uniform Manifold Approximation and Projection (UMAP) plot of the 80742 lung cells that were selected for further analysis. Cells are color coded by the corresponding group. C) UMAP plot showing distinct subpopulations with the cellular identity annotated. D) UMAP plot showing the four major cell types within the lung. E) Box plot showing the proportion of subpopulations of lung epithelial cells among groups. Each dot represents a biological replicate. ABPP, alveolar bipotent progenitor; BASCs, bronchioalveolar stem cells; PNEC, pulmonary neuroendocrine cell. Box plots showing the score of F) Wnt, G) EGFR, and H) Notch signalings among groups via using the gene sets derived from Kyoto Encyclopedia of Genes and Genomes database. I) Box plot showing the NADH regeneration score among groups via using a gene set derived from Gene Set Enrichment Analysis database. J) Box plot showing the lung injury score among groups via using a gene signature confirmed to be significantly associated with the lung function decline in COPD patients. For box plot, center line indicates the median; box limits the upper and lower quartiles; whiskers represent 1.5 × IQR. IQR, interquartile range. ***p < 0.001.

The therapeutic effect of Wnt3a^WG^ EVs was assessed using a murine model of elastase instillation–induced emphysema.^[^ ^6b,c^ ^]^ Seven days after elastase instillation, the mice received an intraperitoneal injection of 200 mg kg^−1^ LiCl per day or an intravenous injection of 2 × 10^9^ particles EVs once every 2 days (**Figure** **5**A). Consistent with a previous report,^[^ ^26^ ^]^ the systemic biodistribution of DiR‐labeled Native EVs and Wnt3a^WG^ EVs were similar after intravenous injection (Figure S9A–C, Supporting Information). To further define the cellular uptake of EVs in the lung, PKH26‐labeled EVs were intravenously injected.^[^ ^25^ ^]^ Both Native EVs and Wnt3a^WG^ EVs could be internalized by Pdpn^+^ alveolar type I (AT1) and Sftpc^+^ alveolar type II (AT2) epithelial cells in elastase‐instilled and sham mice (Figure 5B and Figure S9D, Supporting Information), respectively. While body weight and gross histological morphology were unaffected by EVs, weight loss and hepatocyte consolidation were observed after LiCl administration (Figure 5C and Figure S10, Supporting Information), highlighting its potential systemic toxicity.^[^ ^7c^ ^]^ Lung function analysis showed a reduction in pulmonary compliance, a hallmark of elastase instillation–induced emphysema, after Wnt3a^WG^ EV administration (Figure 5D).^[^ ^6c^ ^]^ Hematoxylin and eosin (H&E) staining further confirmed that 2 × 10^9^ particles Wnt3a^WG^ EVs was sufficient to decrease the enlarged airspace induced by elastase instillation (Figure 5E,F). However, by quantifying the thickness of extracellular matrix and cell number of macrophages (F4/80^+^) and neutrophils (Ly6G^+^) around small airways (Figure S11, Supporting Information), we found that COPD‐specific bronchitis phenotypes generally induced by cigarette smoke did not arise in our elastase‐induced emphysema model, and EV administration did not induce and alter small airway inflammation and fibrosis.^[^ ^27^ ^]^ Only 0.2% of cells were positive for Sftpc and Ki67, markers of proliferating AT2 cells, in the lungs of mice receiving elastase instillation. This was significantly reversed by Wnt3a^WG^ EV treatment (**Figure** **6**G,H and Figure S12, Supporting Information), without altering the ratio of apoptotic cells (Figure S13A,B, Supporting Information). These data suggest that Wnt3a^WG^ EVs promote alveolar epithelial cell proliferation, rather than inhibiting their apoptosis in vivo. Next, we found that Wnt3a^WG^ EV administration restored elastase‐inhibited Wnt3a expression in total lung homogenates (Figure S13C,D, Supporting Information). In addition, since EVs could also reside in the liver, the status of Wnt/*β*‐catenin signaling was also explored.^[^ ^28^ ^]^ We found that both LiCl and Wnt3a^WG^ EVs could significantly increase the percentage of intracellular *β*‐catenin^+^ cells in the liver (Figure S14, Supporting Information). These findings together indicate that Wnt3a^WG^ EVs can activate Wnt/*β*‐catenin signaling in vivo. To determine whether the therapeutic effect was dose‐dependent, 1 × 10^10^ Wnt3a^WG^ EV particles were injected intravenously. Increasing the number of Wnt3a^WG^ EV particles did not result in increased amelioration of the enlarged airspace (Figure S15, Supporting Information). Collectively, these results demonstrate that Wnt3a^WG^ EVs elicit alveolar epithelial regeneration and attenuate lung injury in a murine model of emphysema.

### 2.6 scRNA‐seq‐Based Data Indicates that Wnt3a^WG^ EVs Reinforce Regenerative and Reparative Programs in a Murine Model of Emphysema

scRNA‐seq is a powerful tool that is widely used to understand cellular heterogeneity and provide mechanistic insight into beneficial treatment effects.^[^ ^29^ ^]^ To elucidate the potential mechanisms for the therapeutic function of Wnt3a^WG^ EVs, lung samples were collected and analyzed (Figure 6A). After conducting data integration and quality control, 80742 lung cells were retained (Figure 6B) and their cellular identity was annotated (Figure 6C; Figure S16A, Supporting Information). Using canonical markers (Figure S16B, Supporting Information), the cells were quantitatively clustered into endothelial, epithelial, immune, and stromal cell types (Figure 6D; Figure S16C, Supporting Information). The proportion of cells in particular epithelial cell subpopulations was also determined. There was a reduction in the percentage of AT2 cells, a resident alveolar epithelial cell responsible for epithelium repair and regeneration,^[^ ^5^ ^]^ in response to elastase instillation that increased following Wnt3a^WG^ EV treatment (Figure 6E). To determine whether the signaling required for lung repair and regeneration became activated, a series of regenerative signaling cascades were profiled using Kyoto Encyclopedia of Genes and Genomes (KEGG) data.^[^ ^5^, ^30^ ^]^ The Wnt signaling score was higher after elastase instillation (0.2487) than sham instillation (−0.0081) and Wnt3a^WG^ EV treatment further induced Wnt signaling (0.2593) (Figure 6F; Figure S17A, Supporting Information). In addition, the EGFR signaling score suppressed by elastase (−0.0087) was significantly reversed by Wnt3a^WG^ EVs (−0.0055) (Figure 6G). Pathologically aberrant activation of Notch (Figure 6H) and BMP signaling (Figure S17B, Supporting Information) after injury was also prevented by Wnt3a^WG^ EVs, while Yap and Hedgehog signalings remained unaffected (Figure S17C,D, Supporting Information). These data highlight the important role of Wnt3a^WG^ EVs in promoting beneficial regenerative signaling. To further validate the regenerative function of Wnt3a^WG^ EVs, three different scores were created based on Gene Ontology (GO) terms. Genes associated with nicotinamide adenine dinucleotide (NADH) regeneration were highly enriched in mice receiving elastase instillation (−0.046) and after Wnt3a^WG^ EV treatment (−0.034) (Figure 6I). However, while the score derived from another regenerative GO term was elevated in mice receiving PBS and Wnt3a^WG^ EVs, there was no significant change in the nicotinamide adenine dinucleotide phosphate regeneration score among the three groups (Figure S17E,F, Supporting Information). In addition, we also explored the effect of Wnt3a^WG^ EVs on the proliferation of AT1 cells, which are critical in maintaining the structural integrity of alveoli,^[^ ^5^ ^]^ by calculating the cell proliferation‐associated enrichment scores. We found that the scores were significantly increased in response to elastase instillation, which were unaffected by Wnt3a^WG^ EVs (Figure S18, Supporting Information), further highlighting that AT2 cells can be the major target for Wnt3a^WG^ EVs. The beneficial effect of Wnt3a^WG^ EVs was then confirmed using a gene signature (hereafter termed the lung injury score) that is associated with a decline in COPD patient lung function.^[^ ^31^ ^]^ The lung injury score was significantly higher after elastase instillation (0.0107) than sham (−0.0094) and was significantly decreased by Wnt3a^WG^ EVs (0.0041) (Figure 6J). The status of protease system that plays a prominent role in promoting emphysematous remodeling induced by elastase,^[^ ^32^ ^]^ as well as the antiprotease system, was also investigated by calculating the enrichment scores of indicated gene sets. As expected, the activated protease system induced by elastase instillation could be significantly decreased by Wnt3a^WG^ EVs (Figure S19A,B, Supporting Information), while the increased protease inhibitor complex scores could also be restored (Figure S19C,D, Supporting Information). These findings together revealed that Wnt3a^WG^ EVs reinforce regenerative programs to alleviate lung injury.

## 3 Discussion

This study evaluated gene expression profiles in the lungs of COPD patients and found that Wnt ligand expression was reduced in lung epithelial cells. Given the important role of Wnt/*β*‐catenin signaling in multiple aspects of lung biology, including lung pathogenesis,^[^ ^5^, ^33^ ^]^ Wnt could serve as a useful COPD treatment target. Since Wnt proteins are lipidated and highly hydrophobic, they can only be purified by detergents, limiting their activity both in vitro and in vivo.^[^ ^34^ ^]^ EV‐based delivery of designed cargo has provided more extensive therapeutic options for a number of diseases.^[^ ^35^ ^]^ While different engineering strategies have been designed to deliver hydrophilic proteins, such as cytokines, antibodies, and RNA‐binding proteins, the platforms used to carry therapeutic hydrophobic proteins are rarely reported.^[^ ^14a^ ^]^ Previous studies indicate that Wnt3a is naturally produced on the surface of EVs^[^ ^13^, ^36^ ^]^ and intraarticularly injected Wnt3a‐loaded EVs derived from L‐cells are active and able to induce cartilage repair and regeneration.^[^ ^15^ ^]^ However, the beneficial activity of these EVs largely relies on the stochastic display of Wnt3a on the cell surface, which limits their clinical use in regenerative treatments.^[^ ^15^ ^]^ As a proof of concept, this study provided the first evidence that a hydrophobic Wnt3a protein can be protected by glypicans and anchored to the EV surface in high abundance. These results demonstrate that the combination of WLS‐stimulated Wnt3a secretion and a GPC6^ΔGPI^‐C1C2‐based protective strategy is critical to producing biologically active Wnt3a‐loaded EVs. These findings offer a novel perspective on the use of EVs as a vehicle for the delivery of bioactive hydrophobic proteins.

After performing a detailed characterization and assessment of Wnt3a^WG^ EVs, it was determined whether EV‐based Wnt/*β*‐catenin activation represents an effective therapeutic option for emphysema. LiCl, an agonist of Wnt/*β*‐catenin signaling that inhibits GSK‐3*β* activity, was used as a control. While LiCl treatment was able to partially alleviate airspace enlargement and improve lung morphology, this was accompanied by obvious systemic toxicity, likely because of its effect on other signaling pathways.^[^ ^37^ ^]^ In contrast, body weight and histological morphology were unaffected by Wnt3a^WG^ EV application. EVs from different cell sources have been used to treat fibrotic lung disease by suppressing pro‐fibrotic signaling and delaying the progression of fibrosis.^[^ ^38^ ^]^ Another study showed that mesenchymal stem cell–derived EVs have a beneficial effect on injured lung tissue,^[^ ^39^ ^]^ however, the low quantity of EVs generated from these cells limits their use in translational medicine. The current study showed, for the first time, that EV‐based therapeutic activation of Wnt signaling restores lung architecture and attenuates elastase‐induced emphysema‐associated lung injury by re‐initiating endogenous alveolar regeneration. To further define the mechanisms that mediate the effect of Wnt3a^WG^ EVs, this study used scRNA‐seq, a powerful tool used to identify the cellular and molecular mechanisms of different drug treatments.^[^ ^29^, ^40^ ^]^ Since endogenous regenerative mechanisms are usually impaired in the lungs of COPD patients, Wnt signaling activation is shown to have a therapeutic effect.^[^ ^6^, ^41^ ^]^ The current study found that the therapeutic function of Wnt3a^WG^ EVs was largely due to their reinforcement of pulmonary regeneration mechanisms that are enhanced by Wnt and EGFR signaling, in addition to the regeneration of biological processes. A lung injury score generated from an independent cohort, which was used to flag a significant decline in lung function among patients with COPD, was further adopted to confirm the therapeutic effect of Wnt3a^WG^ EVs.^[^ ^31^ ^]^ Collectively, these findings highlight the central role of regenerative mechanisms in mediating the therapeutic function of Wnt3a^WG^ EVs.

This study has several limitations. First, EVs were isolated and purified by ultra‐centrifugation, which could allow the enrichment of other proteins or aggregates alongside the EVs. Moreover, while ultra‐centrifugation is widely accepted for scientific research, it is not suitable for industrial manufacture. It should also be noted that high EV yields are fundamental for clinical translation.^[^ ^20b^ ^]^ Thus, it will be critical to establish a compatible platform for the large‐scale production of highly purified Wnt3a^WG^ EVs. Second, consistent with previous reports,^[^ ^14^, ^26^ ^]^ most Wnt3a^WG^ EVs accumulate in the liver after intravenous administration. To facilitate increased pulmonary retention of EVs and maximize their benefits, an investigation into different routes of administration is needed to determine the optimal delivery method and frequency. The inhalation of EVs in a nebulized form is proposed as an alternative option.^[^ ^42^ ^]^ Third, while EV‐based therapy is an ideal cell‐free approach, the activation of Wnt‐induced regenerative mechanisms by co‐administering Wnt3a^WG^ EVs and other anti‐inflammatory drugs may provide more extensive COPD treatment options.^[^ ^2^, ^20^, ^43^ ^]^ It will be critical to conduct pre‐clinical studies that test the use of EV‐based Wnt/*β*‐catenin activators and inflammation blockers as a potential dual therapeutic approach. Finally, this study and others have found that transient activation of Wnt/*β*‐catenin does not impact histological morphology or lead to tumorigenesis; however, the role of Wnt signaling in carcinogenesis requires careful assessment to inform the development of future treatments. A growing body of evidence supports the safety and low immunogenicity of cell culture–derived EVs,^[^ ^44^ ^]^ however, long and systemic investigations are still needed before they can be clinically applied.

In summary, EV‐based engineering strategies leverage the natural properties of EVs to efficiently deliver hydrophobic Wnt3a. This study reports, for the first time, a novel cell‐free therapeutic agent, namely Wnt3a^WG^ EVs, that is safe and effective for the treatment of elastase‐induced lung injury. COPD is a respiratory disorder with increasing rates of morbidity and mortality for which there is no available treatment. Wnt3a^WG^ EVs provide a promising candidate for the development of COPD therapies. While the focus of this study is on COPD, the findings reported here may inform the development of treatments for other diseases associated with Wnt signaling and tissue regeneration.

## 4 Experimental Section

### Animals

C57BL/6 mice (wild‐type, male, 8–10 weeks of age) were purchased from the Gempharmatech (China). All experimCental procedures were approved by The Ethics Committee of University of Science and Technology of China (USTCACUC24120122042), and performed in accordance with the Guide for the Care and Use of Laboratory Animals published by the National Institutes of Health of the United States (Eighth Edition, 2011). To establish an emphysema model, mice were anesthetized by intraperitoneal injection of pentobarbital sodium (50 mg kg^−1^); elastase (E7885, Sigma, USA) dissolved in PBS was intratracheally instilled (100 U kg^−1^).^[^ ^6b^ ^]^ Starting from day 7 after elastase instillation, Native EVs (2 × 10^9^ particles per dose) and Wnt3a^WG^ EVs (2 × 10^9^ particles per dose) were injected via the tail vein every other day, meanwhile equal volume of PBS was injected as a control treatment. LiCl (L9650, Sigma) was administrated intraperitoneally at a concentration of 200 mg kg^−1^ every day.

### Cell Culture and Transfection

HEK293T cells were cultured in DMEM (BL304A, Biosharp, China) supplemented with 10% FBS (F7524, Sigma) and 1× penicillin–streptomycin solution (BL505A, Biosharp). A549 cells (CL‐0016, Procell, China) were cultured in Ham's F‐12K (PM150910, Procell) supplemented with 10% FBS (164210‐50, Procell) and 1% penicillin–streptomycin solution (PB180120, Procell). BC1, a human iPSC line established previously,^[^ ^45^ ^]^ was used in the present study. BC1 cells were maintained on Vitronectin (RP01002, Nuwacell, China) coated plates in ncEpic medium (RP01001, Nuwacell), and passaged every 4 days using TrypLE (12 605 028, Thermo Fisher Scientific, USA). Cells were incubated at 37 °C in a humidified atmosphere supplemented with 5% CO_2_. For production of Wnt3a‐loaded EVs, HEK293T cells were seeded into 10 cm dish and transfected with indicated plasmids with Lip2000Transfection Reagent (BL623B, Biosharp) according to the manufacturer's instructions. For cohort#1, cells were transfected with Wnt3a (15 µg), Wnt3a‐T2A‐WLS (15 µg), and Wnt3a‐T2A‐WLS‐CD81 (15 µg) plasmids, respectively. For cohort#2, cells were transfected with Wnt3a‐T2A‐WLS (10 µg) plasmid in the presence of GPC4^ΔGPI^ (10 µg), GPC6^ΔGPI^ (10 µg), GPC4^ΔGPI^‐C1C2 (10 µg), and GPC6^ΔGPI^‐C1C2 (10 µg) plasmids, respectively. Conditioned medium was collected for three consecutive days post‐transfection and may be stored for later purification.

### Isolation and Characterization of EVs

EVs were isolated from conditioned medium (CM) by sequential ultracentrifugation method.^[^ ^46^ ^]^ CM was subjected to centrifugation steps at 300 × *g* for 10 min, 2000 × *g* for 10 min, and 100 000 × *g* for 70 min using the Optima MAX‐XP Ultracentrifuge (Beckman, USA). Supernatants were aspirated; pellets were then washed with PBS and ultra‐centrifuged at 100 000 × *g* for 70 min. Purified EVs were stored at −80 °C. The particle concentration and size distribution of EVs were measured by NTA using a ZetaView PMX 110 analyzer (Particle Metrix, Meerbusch, Germany) equipped with a ZetaView 8.04.02 SP2 software. For TEM analysis, EVs were loaded onto a carbon‐coated electron microscopy grid and stained with 2% uranyl acetate for 1.5 min. Excessive liquid was removed, and images were captured using an electron microscope (Tecnai F12, FEI, USA).

### Luciferase Reporter Assay

To validate the bioactivity of Wnt3a delivered via EVs, a luciferase reporter assay was applied. HEK293T cells (1 × 10^5^ cells per well) were seeded into 24‐well plate. Cells were transfected with a mixture (30:1) of TOPFlash luciferase reporter (a gift from Professor Yuyong Tao) and pRL‐TK reporter (Promega, USA) plasmids with Lip2000Transfection Reagent. After treatment with 48 h, cells were collected and analyzed using a Dual Luciferase Reporter Assay Kit (DL101‐01, Vazyme, China) according to the manufacturer's instructions. The absorbance was determined at 560 and 465 nm using a SpectraMax iD5 (Molecular Devices, Germany) equipped with a SoftMax Pro 7 software.

### Cell Growth and Apoptosis Assay

To establish a human alveolar epithelial cell injury model, A549 cells were seeded and cultured overnight at 37 °C. Cells were then treated with LPS (BS904, Biosharp) and further incubated for 48 h before adding the indicated treatments. For cell growth assay, cells were seeded into 96‐well plate. After treatment, 10 µL CCK‐8 solution (C0038, Beyotime, China) was added into each well. After an incubation for 2 h, the absorbance was determined at 450 nm using a SpectraMax iD5. For cell apoptosis assay, cells were seeded into a 6‐well plate. After treatment, cells were collected and stained using the FITC Annexin V Apoptosis Detection Kit (556 547, BD Biosciences, USA). Samples were analyzed using a BD LSRFortessa flow cytometer (BD Biosciences). Data were processed using FlowJo software (BD Biosciences).

### DiR‐Labeled EVs

To determine the biodistribution of EVs after intravenous injection, Native EVs and Wnt3a^WG^ EVs were incubated with DiR fluorescent dye (40757ES25, Yeasen, China) at concentration of 100 µg mL^−1^ for 30 min at 37 °C. Labeled EVs were washed with PBS and ultra‐centrifuged at 120 000 × *g* for 35 min to remove the free dye. For real‐time imaging, mice were injected via the tail vein with DiR‐labeled EVs (2 × 10^9^ particles); images were captured at 0, 24, and 48 h using a IVIS Lumina III system (PerkinElmer, USA).

### PKH26‐Labeled EVs

To explore the cellular uptake of EVs, Native EVs and Wnt3a^WG^ EVs were labeled with PKH26 (MINI26, Sigma) as reported previously.^[^ ^25^ ^]^ In brief, a total of 250 µL EV suspension was prepared by mixing with Diluent C; the Dye solution was prepared by adding 1 µL of PKH26 solution to 250 µL of Diluent C. The EV suspension was then added to the Dye solution, and mixed immediately by pipetting. The staining was stopped via adding an equal volume of FBS; the mixture was then ultra‐centrifuged at 120 000 × *g* for 35 min to remove the free dye. The pellet of labeled EVs was washed with PBS and ultra‐centrifuged at 120 000 × *g* for 35 min. For in vitro experiments, cells were added with PKH26‐labeled EVs at a concentration of 2 × 10^9^ particles per mL and further incubated for 6 h. Cells were fixed with 4% PFA and stained with DAPI; images were then captured using a microscope (DMi8, Leica, Germany). For in vivo experiments, mice were injected via tail vein with PKH26‐labeled EVs (2 × 10^9^ particles). After administration with 12 h, lung samples were collected and fixed with 4% PFA. These samples were then embedded with OCT compound (Sakura, USA) and sectioned. Tissue sections were stained with Sftpc (ab211326, Abcam, USA) and Pdpn (sc‐376962, Santa Cruz, USA) antibodies, respectively; images were then captured using a microscope (DMi8, Leica).

### Immunohistochemistry and Immunofluorescence

Tissues were fixed with 4% PFA, embedded in paraffin, and sectioned. H&E (G1005, Servicebio, China) and Masson's trichrome (G1006, Servicebio) staining were conducted according to the manufacturer's instructions, respectively. For immunofluorescence analysis, deparaffinized slices were incubated with an Improved Citrate Antigen Retrieval Solution (P0083, Beyotime), and stained with primary antibodies against Sftpc (ab211326, Abcam, USA), Ki67 (GB121141, Servicebio), *β*‐catenin (8480S, Cell Signaling Technology, USA), and DAPI (C1002, Beyotime). Tunel staining (G1502, Servicebio) was performed according to the manufacturer's instructions. For immunohistochemistry staining of macrophages and neutrophils, deparaffinized slices were incubated with F4/80 (GB113373, Servicebio) and Ly6G (GB11229, Servicebio) antibodies, respectively. For immunocytochemistry analysis, cells were fixed with 4% PFA and stained with primary antibodies against BRACHYURY (ab209665, Abcam) and Oct4 (sc‐5279, Santa Cruz).

### qRT‐PCR

Total RNA was isolated from cells and tissues using TRIzol (Invitrogen, USA). cDNA libraries were synthesized through using a Reverse Transcription Reagent kit (Takara, Japan). Samples were then used for real‐time PCR with SYBR Green mix (Takara), and results were normalized to the expression of house‐keeping genes including *β*‐actin and Gapdh, respectively. Primers were obtained from Tsingke (China), and sequences are listed in Table S1, Supporting Information. Data were analyzed by using a comparative Ct method.

### Western Blotting

Cells and EVs were lysed using a lysis buffer (Cell Signaling Technology, USA) supplemented with protease inhibitors (BL612A, Biosharp) at 4 °C for 30 min. Protein concentration was then quantified using a BCA protein assay kit (BL521A, Biosharp). Western blotting was performed as previously described;^[^ ^47^ ^]^ bands were captured using the FluorChem M system (ProteinSimple, USA). Primary antibodies against *β*‐catenin (8480S), Wnt3a (2721S), Alix (2171S), and Calnexin (2433S) were purchased from Cell Signaling Technology. Primary antibodies against CD63 (sc‐5275) and Oct4 (sc‐5279) were purchased from Santa Cruz. Primary antibodies against *β*‐actin (66009‐1‐Ig) and GAPDH (10494‐1‐AP) were purchased from Proteintech (China). Primary antibody against BRACHYURY was purchased from Abcam. Primary antibody against TSG101 (GB11618) was purchased from Servicebio.

### Mouse Single‐Cell RNA Sequencing Data Generation

Lungs were isolated from mice and dissected into small pieces, then stored at GEXSCOPE Tissue Preservation Solution (Singleron Biotechnologies, China). All samples were digested at 37 °C for 15 min. After being filtered using a 40 micron sterile strainer, cell suspensions were collected and centrifuged at 500 × *g* for 5 min at 4 °C. The pellets resuspended in PBS were incubated with GEXSCOPE Red Blood Cell Lysis Buffer (Singleron Biotechnologies) at room temperature for 10 min to remove red blood cells. After centrifuge at 500 × *g* for 5 min at 4 °C, single‐cell suspensions were counted using a TC20 Automated Cell Counter (Bio‐Rad, USA). Single‐cell suspensions were loaded onto microfluidic devices. Libraries were then constructed according to the Singleron GEXSCOPETM protocol by using the GEXSCOPETM Single‐Cell RNA Library Kit (Singleron Biotechnologies). Libraries were diluted to 4 nm and pooled for sequencing, respectively. Pools were sequenced using an Illumina HiSeq X platform.

### Data Processing

Raw data was processed via using CeleScope pipeline (version 1.10.0) with default parameters. Raw reads were then aligned to the mouse reference genome GRCm38 (version 92). Additionally, single‐cell RNA sequencing data of lungs from C57BL/6 mice without injury was retrieved from two independent cohorts.^[^ ^48^ ^]^ Preliminary counts were selected for downstream quality control. Three criterions were adopted to filter cells with poor quality: 1) the number of detected genes fewer than 200 and greater than 6000; 2) the total UMI counts greater than 55 000; 3) the proportion of mitochondrial gene counts greater than 15. Cells identified as doublets were removed by using DoubletDetection (version 4.2). Eventually, a total of 80 742 cells were retained for subsequent analysis.

### Data Integration

The expression values were normalized to 10 000 transcripts per cell and log‐transformed using Scanpy (version 1.9.1).^[^ ^49^ ^]^ The “scanpy.pp.highly_variable_genes” function was used to detect highly variable genes; top 1500 genes with the highest frequencies were selected as the most variable genes. Cells with such informative genes were then scaled with the regression of total UMI counts and percent mitochondrial transcripts by using the “scanpy.pp.regress_out” and “scanpy.pp.scale” functions. To correct the batch effect of different datasets, Harmony algorithm was performed.^[^ ^50^ ^]^ The scaled matrix was used to compute the Principal Component Analysis, which was further modified to Harmony matrix. Sample and sequencing platform were considered batches for correction with theta set as 2.5 and 1.5, respectively.

### Dimensionality Reduction and Clustering

The batch‐corrected matrix was employed to construct the nearest neighbor graph, and Leiden algorithm was used to find clusters. Uniform Manifold Approximation and Projection (UMAP) was conducted for the dimensionality reduction and visualization. The “scanpy.tl.leiden” function was adopted to cluster all cells and distinguish four major cell types by the expression of Pecam1, Epcam, Ptprc, and Col1a2. Single cells expressing two sets of classical markers of major cell types were labeled as doublets and removed. Then, independent clustering was performed in each major cell type with a higher resolution using the method described above. These four major cell types were further divided into 35 clusters representing different cell types within major cell lineages.

### Calculation of Signaling Score

Scoring for the enrichment of gene sets was calculated using the “scanpy.tl.score_genes” function. The signaling score is the average expression of a set of genes subtracted with the average expression of a reference set of genes, which is randomly sampled from all genes. Gene lists were collected as following: 1) downstream target genes of the signaling involved in the lung regeneration were retrieved from KEGG;^[^ ^51^ ^]^ 2) GO terms were obtained from Gene Set Enrichment Analysis;^[^ ^52^ ^]^ 3) a signature consisted of 120 genes, which the higher expression is significantly associated with the lung function decline in COPD patients, was adopted.^[^ ^31^ ^]^ Statistical significance was calculated using Wilcoxon two‐sided rank‐sum test.

### Human Single‐Cell RNA Sequencing Data

Single‐cell RNA sequencing data generated from human lung tissues of six COPD patients and three never‐smokers were obtained from the Gene Expression Omnibus (GSE173896). The initial gene‐barcode matrix was analyzed using the Seurat (version 4.0) workflow.^[^ ^29^ ^]^

### Statistical Analysis

Data are represented as mean ± standard deviation (SD). Two groups were compared using two‐tailed Student's *t*‐test; multiple groups were compared using One‐way ANOVA and Tukey's multiple comparison test. *P* value < 0.05 was considered significant. Statistical analyses were conducted using Prism version 7.0 (GraphPad Software).

## Conflict of Interest

L.G., D.M., L.C., and S.L. are inventors on a patent application submitted by the University of Science and Technology of China. All other authors declare no conflict of interest.

## Supporting information

Supporting Information

## Data Availability

The data that support the findings of this study are available from the corresponding author upon reasonable request.
