# Supplementary material for: Wnt3a‐Loaded Extracellular Vesicles Promote Alveolar Epithelial Regeneration after Lung Injury
Source: Adv Sci (Weinh). 2023 Apr 18;10(18):2206606. doi: 10.1002/advs.202206606 (PMC10288279; doi:10.1002/advs.202206606)
Supplement: Supplementary file 1 — Supporting Information [file ADVS-10-2206606-s001.pdf]

ADVANCED SCIENCE
Open Access

Supporting Information

for *Adv. Sci.*, DOI 10.1002/advs.202206606

Wnt3a-Loaded Extracellular Vesicles Promote Alveolar Epithelial Regeneration after Lung Injury

Lei Gao, Yongping Sun, Xinye Zhang, Ding Ma, An Xie, Enyu Wang, Linzhao Cheng and Senquan Liu**

Supporting Information

Wnt3a-loaded extracellular vesicles promote alveolar epithelial regeneration after lung injury

Lei Gao[#], Yongping Sun[#], Xinye Zhang[#], Ding Ma, An Xie, Enyu Wang, Linzhao Cheng, and Senquan Liu**

L. Gao, D. Ma, L. Cheng, S. Liu
Department of Hematology, The First Affiliated Hospital of USTC, Division of Life Sciences and Medicine, University of Science and Technology of China, Hefei, Anhui, 230027, China

L. Gao, D. Ma, A. Xie, E. Wang, L. Cheng, S. Liu
Blood and Cell Therapy Institute, Anhui Provincial Key Laboratory of Blood Research and Applications, University of Science and Technology of China, Hefei, Anhui, 230027, China

L. Gao, Y. Sun, X. Zhang, L. Cheng, S. Liu
School of Basic Medical Sciences, Division of Life Sciences and Medicine, University of Science and Technology of China, Hefei, Anhui, 230027, China

*Correspondence
E-mail: liusenquan1988@ustc.edu.cn; lzcheng@ustc.edu.cn

[#]These authors contributed equally

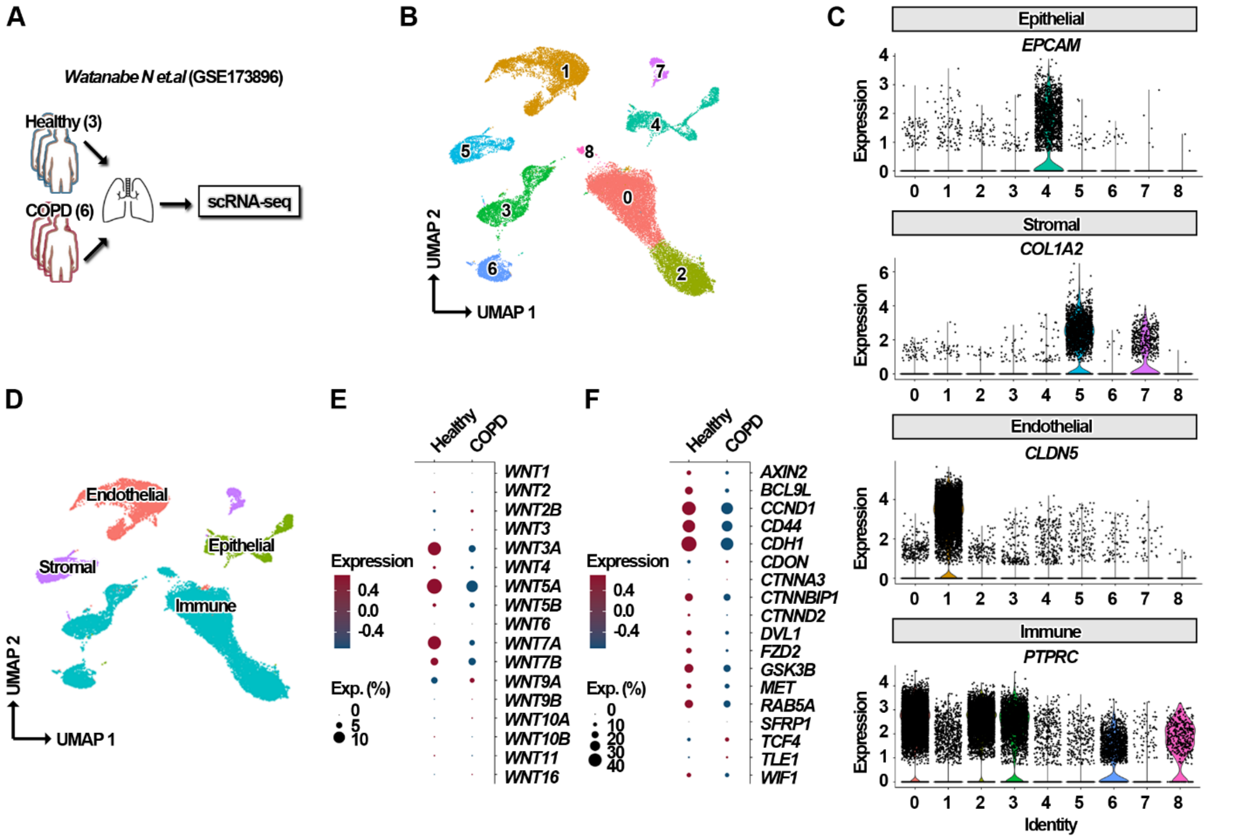

Figure S1. The expression of *WNT3A* is reduced in lung epithelial cell from patients with COPD. A) Schematic of the experimental design. COPD, chronic obstructive pulmonary disease; scRNA-seq, single-cell RNA sequencing. B) Uniform manifold approximation and projection (UMAP) plot revealed that human lungs contained 9 distinct clusters. C) Violin plot showing the expression of *EPCAM*, *CLDN5*, *COL1A2*, and *PTPRC* across 9 clusters in human lungs. D) UMAP plot showing cluster distribution of four major cell types in human lungs. Dot plot analysis of the ligands (E) and the downstream target genes of Wnt signaling (F) in epithelial cells of healthy volunteers and COPD patients.

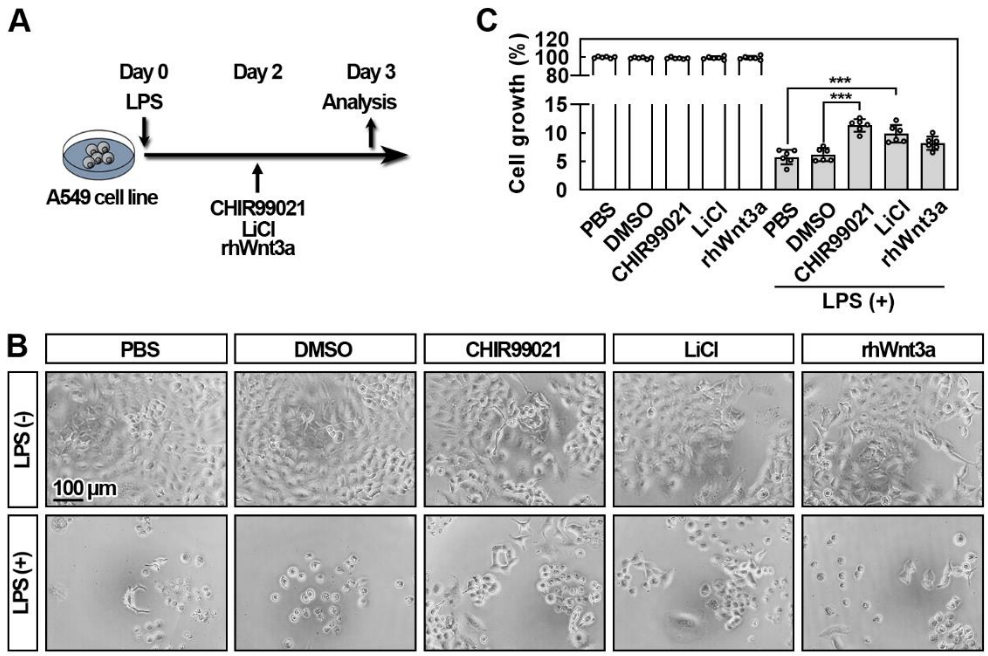

Figure S2. Activating Wnt/β-catenin signaling promotes cell growth in a human alveolar epithelial cell injury model. A) Schematic of the experimental design. A549 cell line was treated with LPS (50 μg/mL) for two days, and the treatments including CHIR99021 (1 μM), LiCl (5 mM), and rhWnt3a (200 ng/mL) were then added for another day. Cells were then photographed (B) and analyzed with CCK-8 assay (C), respectively. Data are shown as mean ± SD from two independent experiments. ***, $p < 0.001$.

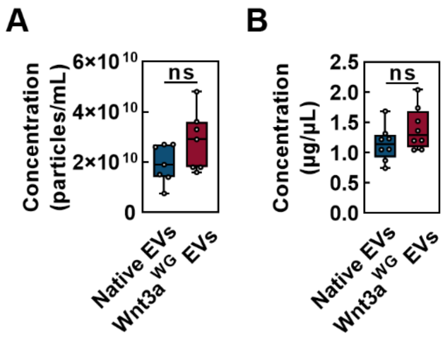

Figure S3. Quantification of EVs. The concentration of Native EVs and Wnt3a^{WG} EVs was determined by NTA (A) and BCA (B), respectively. For A, data are shown as mean ± SD from seven biological replicates. For B, data are shown as mean ± SD from eight biological replicates. ns, not significant.

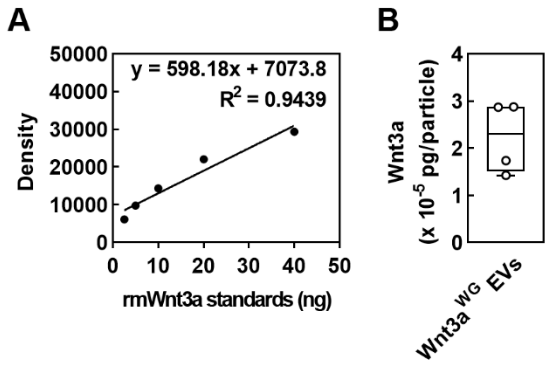

Figure S4. Quantification of Wnt3a protein in Wnt3a^{WG} EVs. A) A representative standard curve was generated based on the different amounts of recombinant murine Wnt3a (rmWnt3a) and the band density as determined by Western Blot (WB). Three independent experiments were performed. B) The amount of Wnt3a on per Wnt3a^{WG} EVs was calculated from quantitative blots and the corresponding concentration of particles as detected by NTA. Data are shown as mean ± SD from four biological replicates.

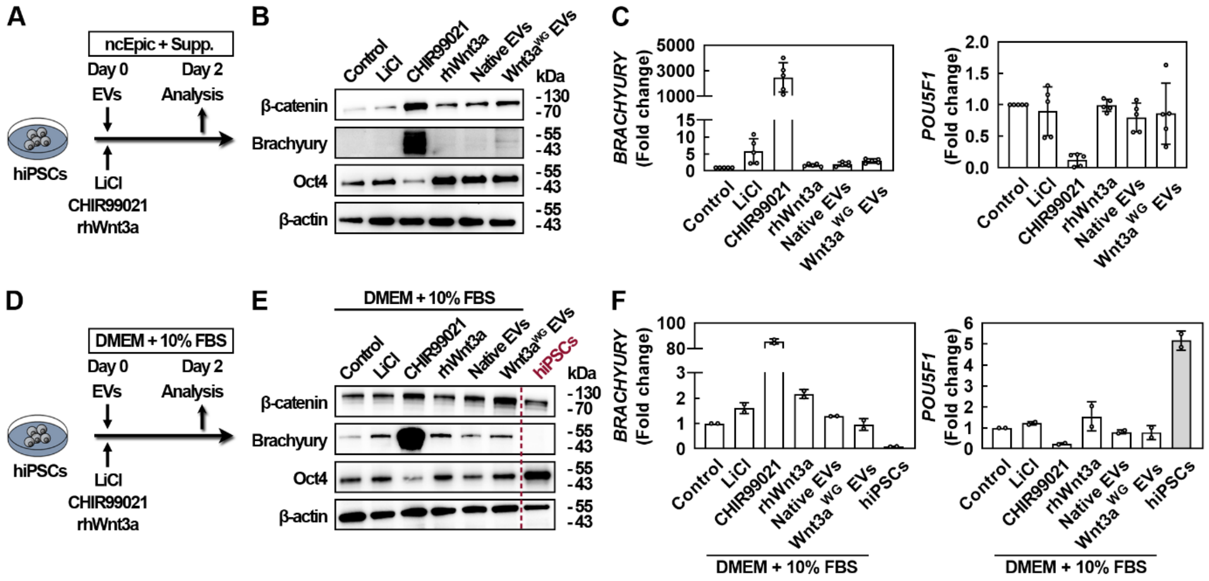

Figure S5. Neither rhWnt3a nor Wnt3a^{WG} EVs can promote the mesoderm differentiation of hiPSCs in the context of setting conditions. A) Schematic of the experimental design. hiPSCs were treated with LiCl (5 mM), CHIR99021 (6 μM), rhWnt3a (100 ng/mL), Native EVs (2 x 10^9 particles/mL), and Wnt3a^{WG} EVs (2 x 10^9 particles/mL) in ncEpic with supplement (Supp.). Cells were then collected for WB (B) and qRT-PCR (C) analyses. For B, representative images of three independent experiments are shown. For C, data are shown as mean ± SD from five independent experiments. D) Schematic of the experimental design. hiPSCs were treated with LiCl (5 mM), CHIR99021 (6 μM), rhWnt3a (100 ng/mL), Native EVs (2 x 10^9 particles/mL), and Wnt3a^{WG} EVs (2 x 10^9 particles/mL) in DMEM supplemented with 10% FBS. Cells were then collected for WB (E) and qRT-PCR (F) analyses. For E, representative images of three independent experiments are shown. For F, data are shown as mean ± SD from two independent experiments.

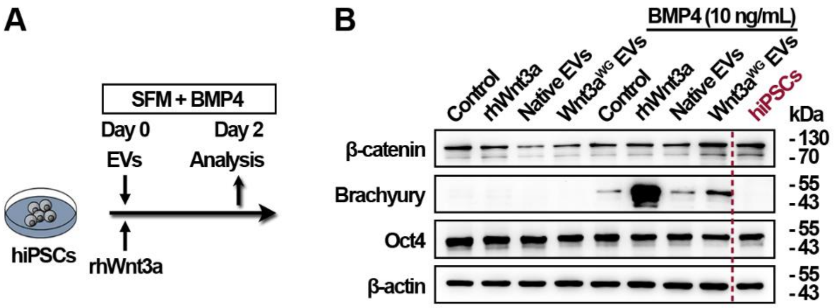

Figure S6. Wnt3a^{WG} EVs promote the mesoderm differentiation of hiPSCs in the context of setting condition. A) Schematic of the experimental design. B) hiPSCs were treated with rhWnt3a (40 ng/mL), Native EVs (2 x 10^9 particles/mL), and Wnt3a^{WG} EVs (2 x 10^9 particles/mL) in the absence or presence of BMP4 (10 ng/mL) during mesoderm differentiation induced by SFM medium for 48 h. Representative images of three independent experiments are shown.

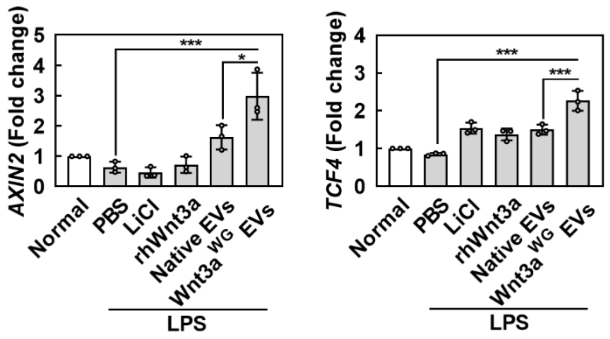

Figure S7. The mRNA abundance of *AXIN2* and *TCF4* in A549 cells treated as indicated in a human alveolar epithelial cell injury model. Data are shown as mean ± SD from three independent experiments. *, $p < 0.05$; ***, $p < 0.001$.

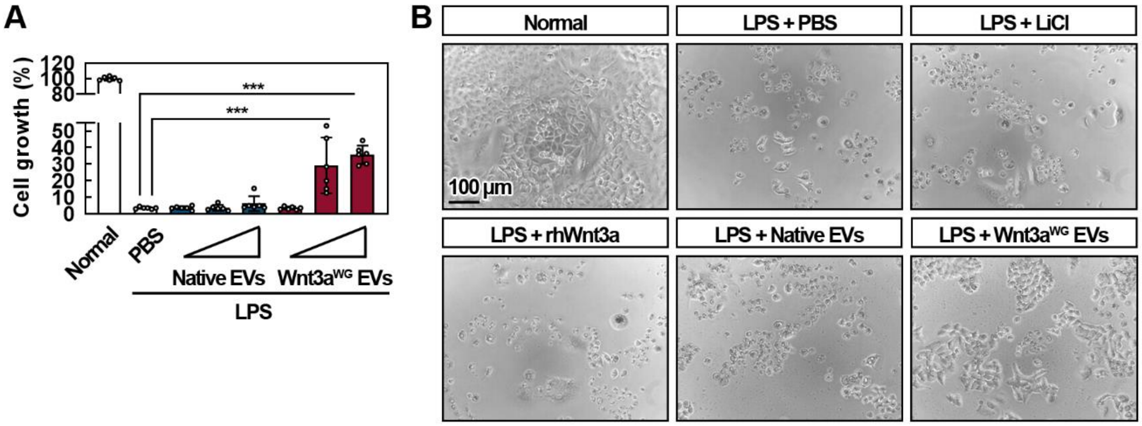

Figure S8. Wnt3a^{WG} EVs promote cell growth in a human alveolar epithelial cell injury model. A) A549 cells were treated with LPS (50 μg/mL) for two days; Native EVs and Wnt3a^{WG} EVs at concentrations of 2 x 10^8, 2 x 10^9, and 4 x 10^9 particles/mL were added. After treatment for two days, cells were then analyzed with CCK-8 assay. Data are shown as mean ± SD from two independent experiments. ***, $p < 0.001$. B) Representative bright filed images of A549 cells treated as indicated. Two independent experiments were performed.

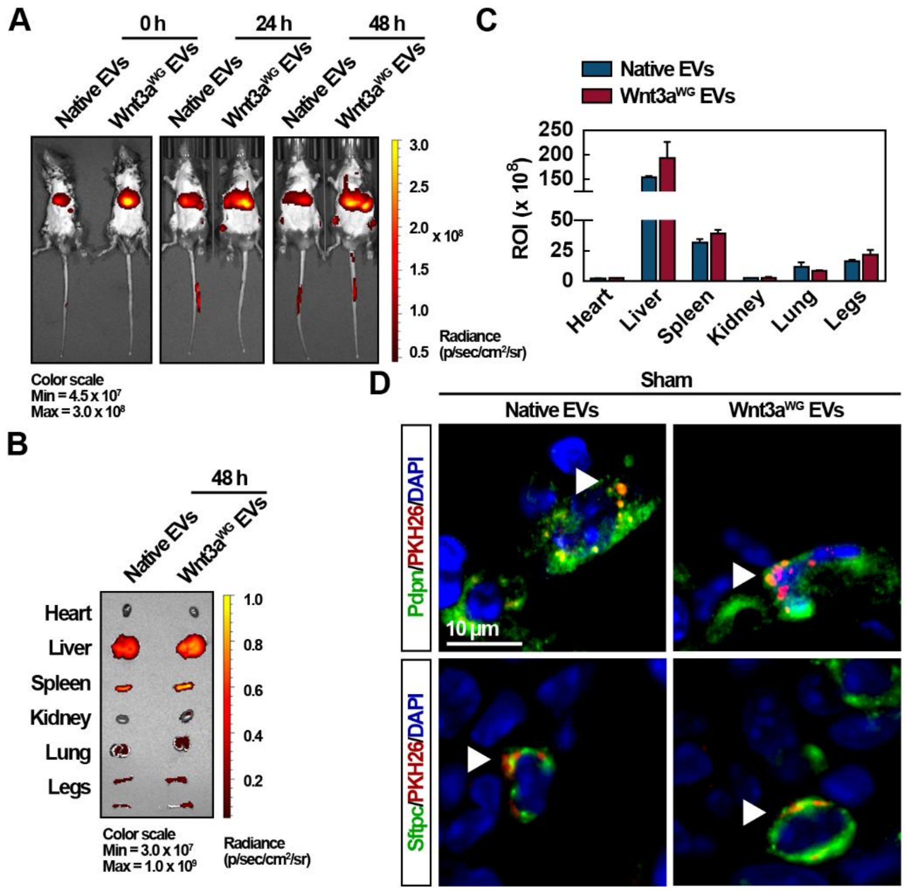

Figure S9. Biodistribution of EVs after intravenous administration. A) Representative real-time *in vivo* images of mice receiving intravenous injection of DiR-labeled EVs. B) Representative images of main organs collected from (A). Organs were annotated on the left side of the panel. C) Quantification of the organ biodistribution profile from *ex vivo* imaging of DiR EV-treated mice. Individual regions of interest (ROI) were obtained for each organ to obtain the respective fluorescence signals. Data are shown as mean ± SD from at least two biological replicates. D) Representative images of immunofluorescent analysis for Pdpn and Sftpc in lung tissue sections from sham mice after intravenously administration with PKH26-labelled EVs (red), respectively. Tissue sections from four biological replicates per group were analysed.

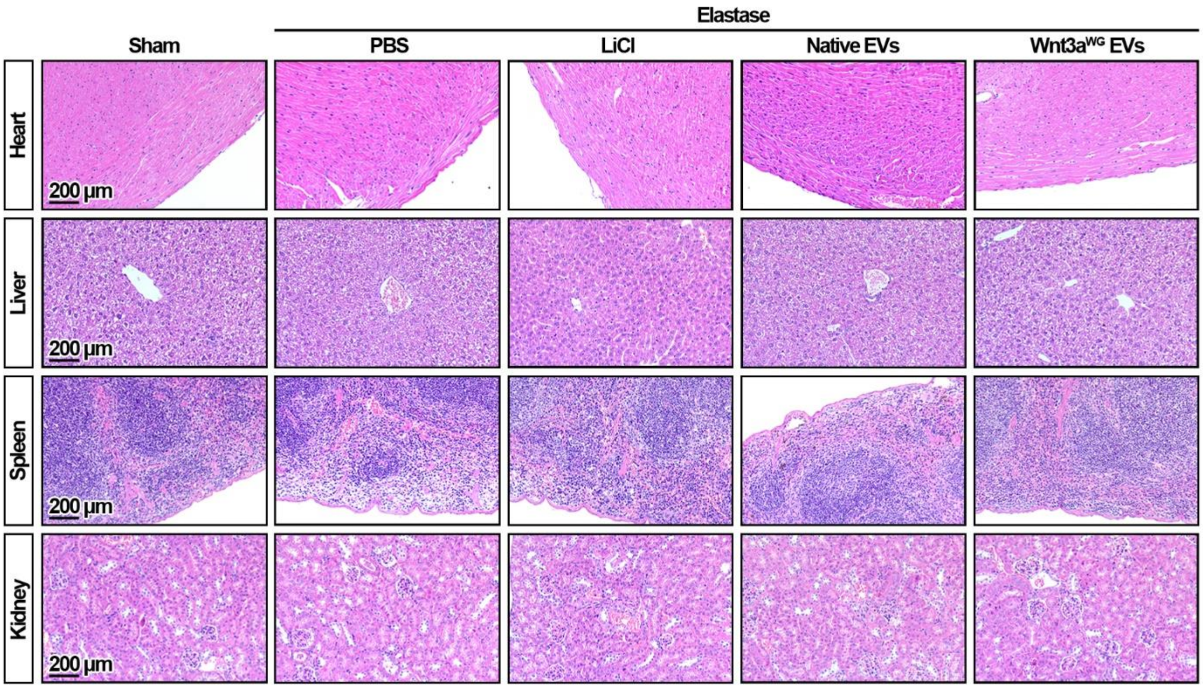

Figure S10. Systemic impact of Wnt3a^{WG} EVs in a murine model of emphysema. Representative images of H&E-stained heart, liver, spleen, and kidney sections from mice treated as indicated. Tissue sections from three biological replicates per group were analysed.

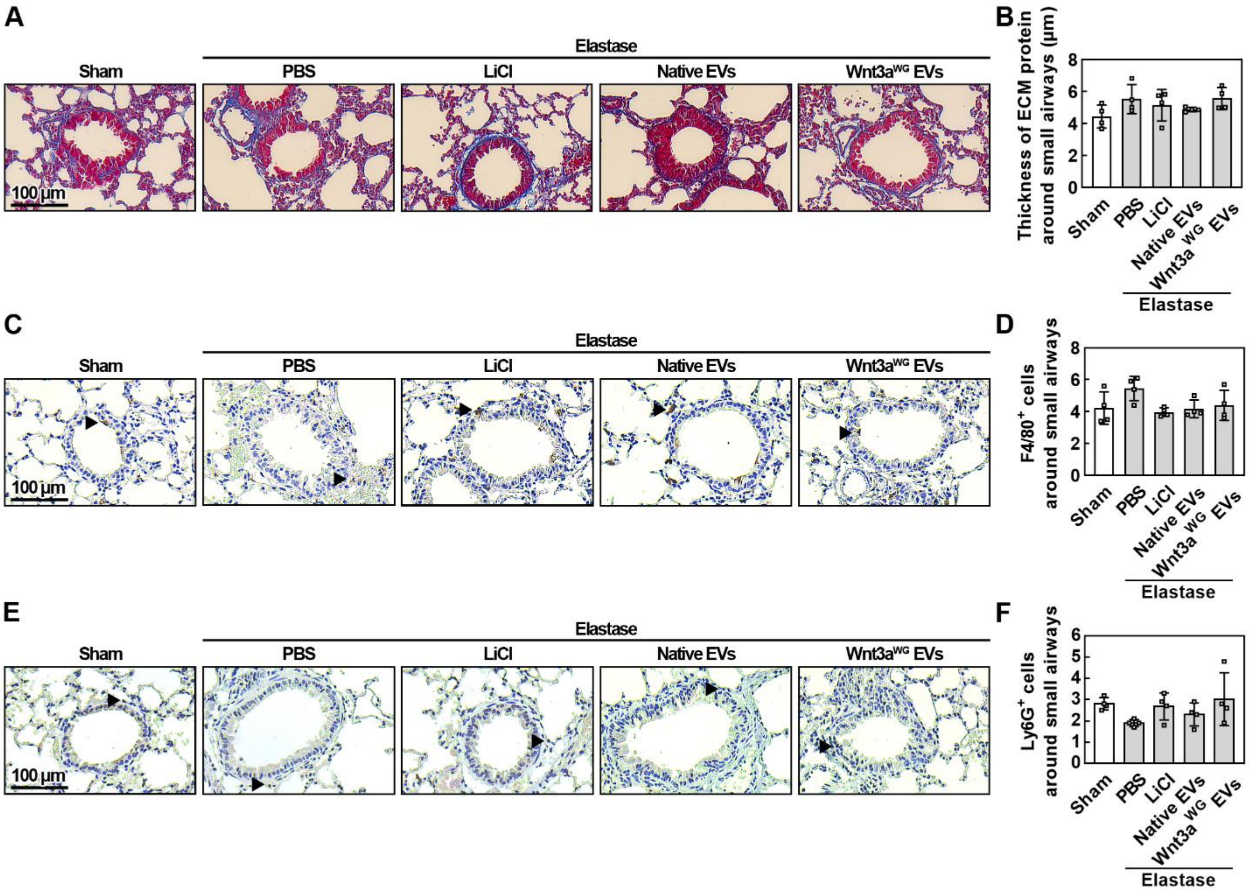

Figure S11. The effect of Wnt3a^{WG} EVs on lung fibsis and inflammation in a murine model of emphysema. A) Representative images of Masson's trichrome-stained lung tissue sections from mice treated as indicated. B) Quantification of the thickness of extracellular matrix (ECM) protein around small airways. Data are shown as mean ± SD from four biological replicates. C) Representative images of IHC analysis for F4/80 (macrophages) in lung tissue sections from mice treated as indicated. D) Quantification of F4/80^{+} cells around small airways. Data are shown as mean ± SD from four biological replicates. E) Representative images of IHC analysis for Ly6G (neutrophils) in lung tissue sections from mice treated as indicated. F) Quantification of Ly6G^{+} cells around small airways. Data are shown as mean ± SD from four biological replicates.

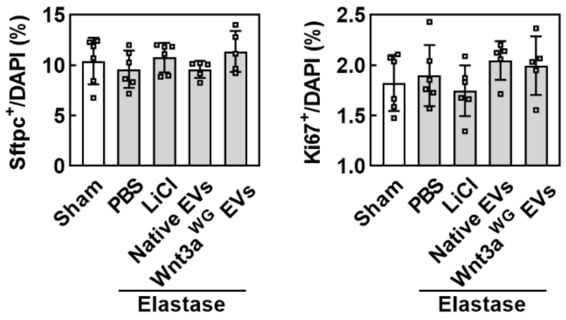

Figure S12. Quantification of Sftpc^{+} and Ki67^{+} cells after treatment with Wnt3a^{WG} EVs in a murine model of emphysema. Data are shown as mean ± SD from at least five biological replicates.

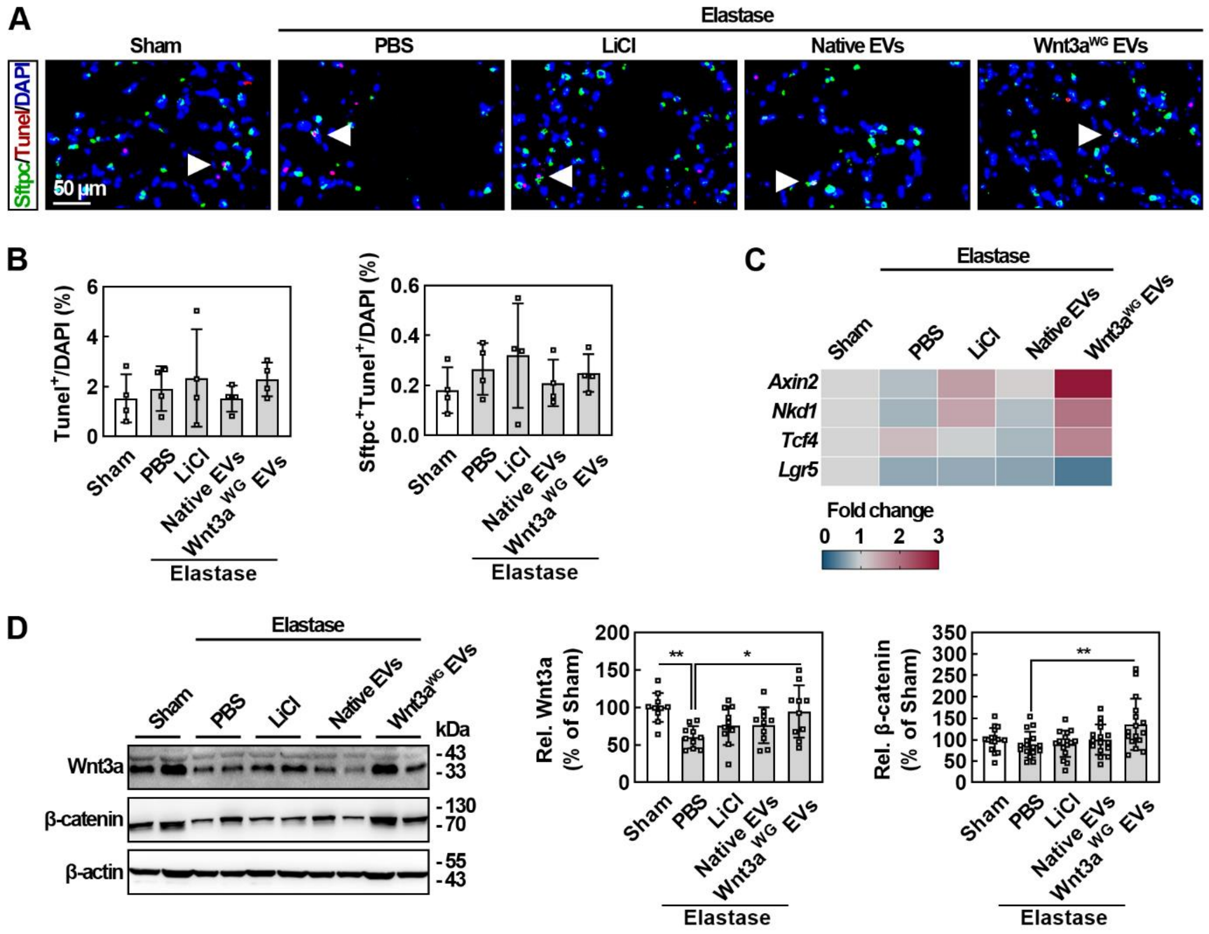

Figure S13. Wnt3a^{WG} EVs partially activate Wnt signaling in a murine model of emphysema. A) Representative images of immunofluorescent analysis for Sftpc and Tunel in lung tissue sections from mice treated as indicated. B) Quantification of apoptotic cells (Tunel^{+}) and alveolar stem cells (Sftpc^{+}) positive for Tunel. Data are shown as mean ± SD from four biological replicates. C) Heat map of mRNA abundance of Wnt signaling target genes determined by qRT-PCR from lungs of mice treated as indicated. D) WB analysis of Wnt3a and β-catenin in whole lungs collected from mice treated as indicated. Representative images of at least three independent experiments are shown. Data are shown as mean ± SD from six biological replicates. *, $p < 0.05$; **, $p < 0.01$.

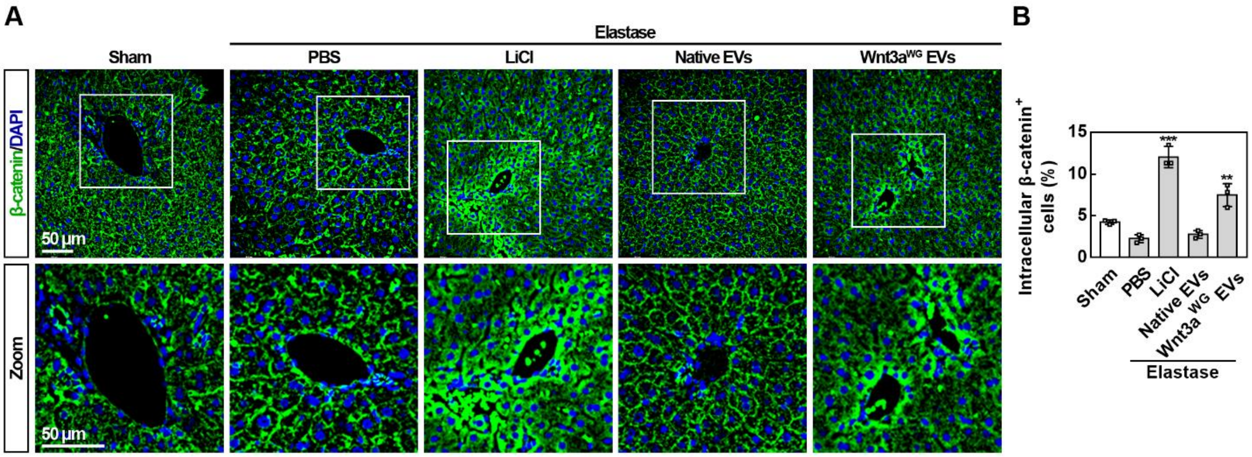

Figure S14. The effect of Wnt3a^{WG} EVs on Wnt/β-catenin signaling in liver in a murine model of emphysema. Representative images of immunofluorescent analysis for β-catenin (A) and quantification of intracellular β-catenin^{+} cells (B) in liver tissue sections from mice treated as indicated. Data are shown as mean ± SD from three biological replicates. *, significantly different from Sham; **, $p < 0.01$; ***, $p < 0.001$.

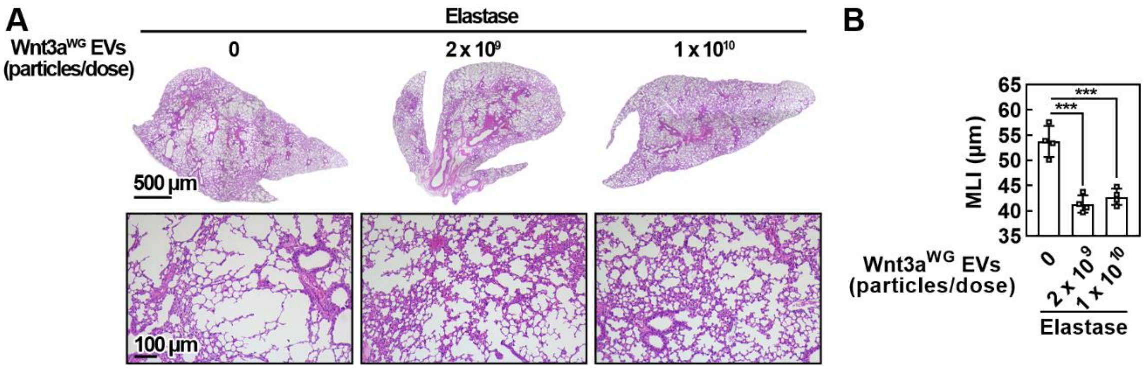

Figure S15. Wnt3a^{WG} EVs restore enlarged airspace in a murine model of emphysema. A) Representative images of H&E-stained lung tissue sections. B) Quantification of airspace enlargement as MLI. Data are shown as mean ± SD from four biological replicates. ***, $p < 0.001$.

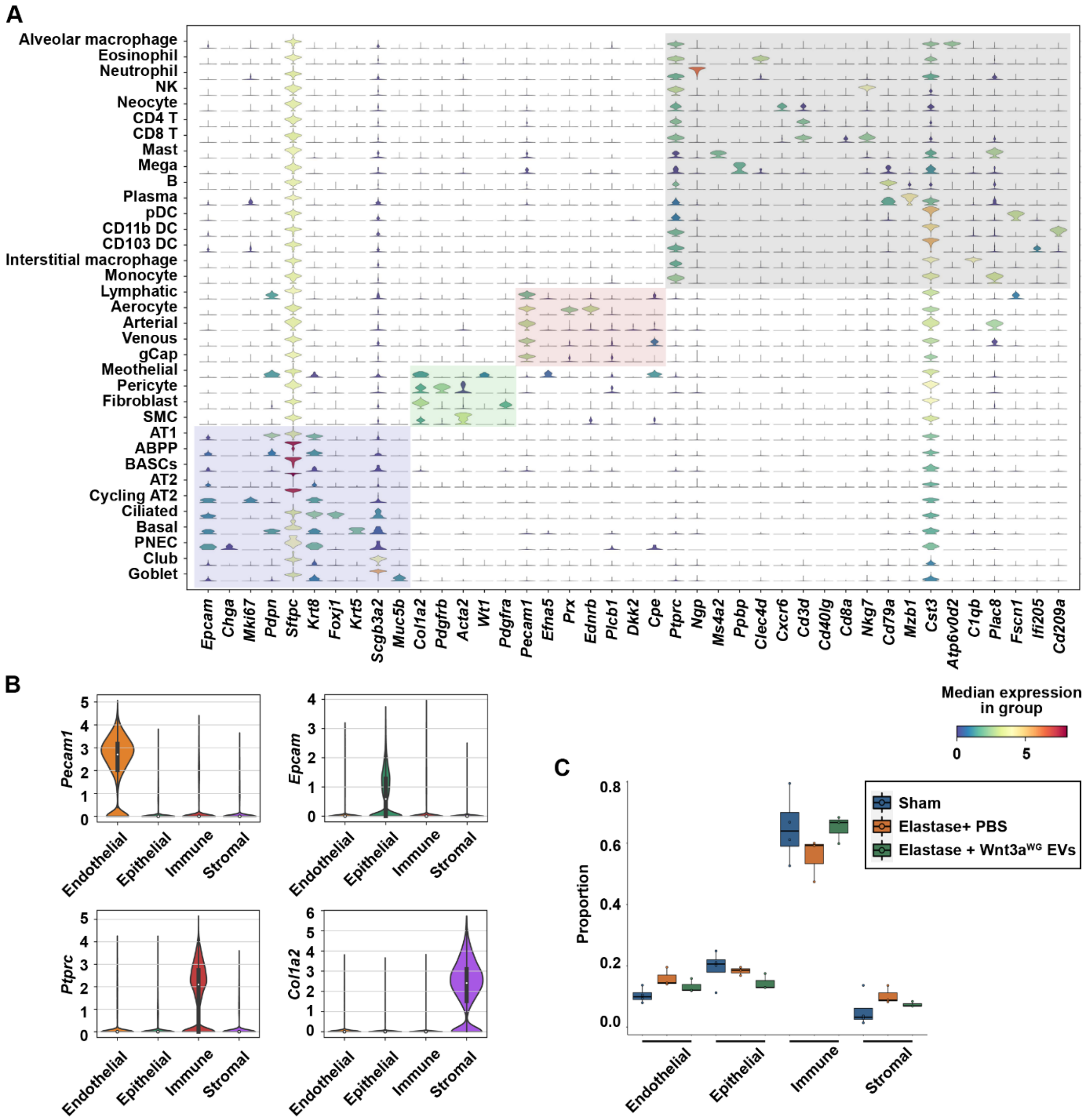

Figure S16. Canonical markers-based analysis. A) Violin plot showing the expression of marker genes for each cell type. NK, natural killer; pDC, plasmacytoid dendritic cells; gCap, general capillary; SMC, smooth muscle cell; ABPP, alveolar bipotent progenitor; BASCs, bronchioalveolar stem cells; PNEC, pulmonary neuroendocrine cell. B) Violin plots verifying the expression of *Pecam1*, *Epcam*, *Ptprc*, and *Col1a2* among four major cell types in the lung. C) Box plot showing the proportion of four major cell types in the lung. Each dot represents a biological replicate.

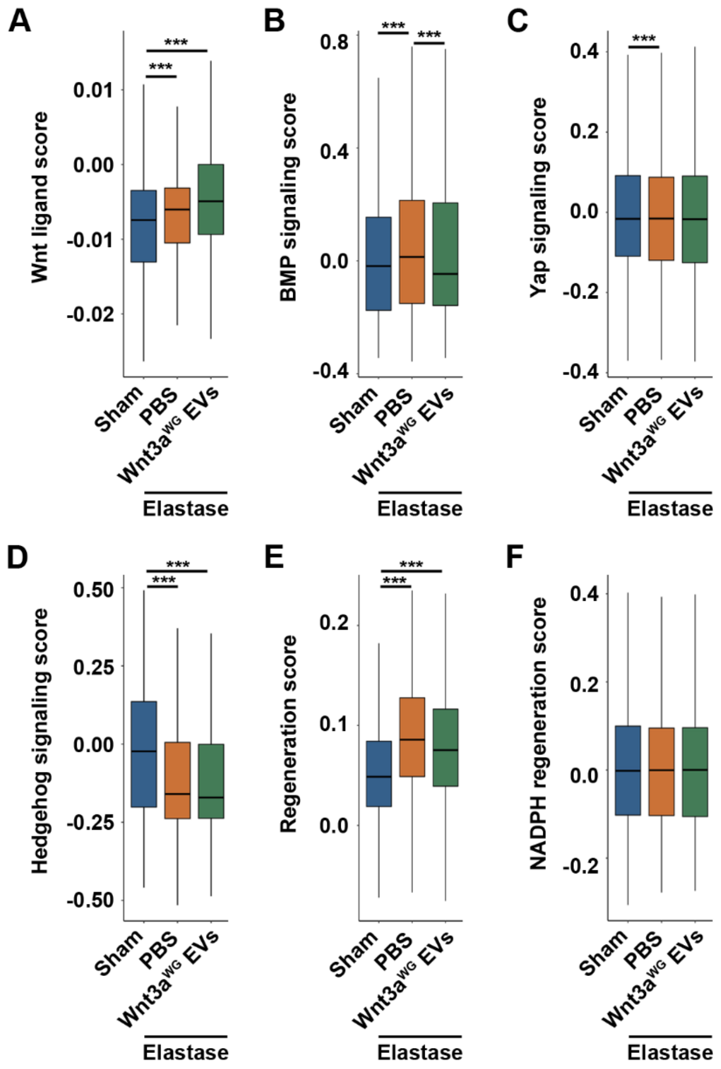

Figure S17. The status of signalings involved in the tissue regeneration process. A) Box plot showing the Wnt ligand score among groups. Box plots showing the scores of BMP (B), Yap (C), and Hedgehog (D) signalings among groups via using the gene sets derived from Kyoto Encyclopedia of Genes and Genomes database. Box plots showing the regeneration (E) and NADPH regeneration (F) scores among groups via using a gene set derived from Gene Set Enrichment Analysis database. For box plot, the center line indicates the median; the box limits the upper and lower quartiles; the whiskers represent 1.5 × IQR. IQR, interquartile range. ***, $p < 0.001$.

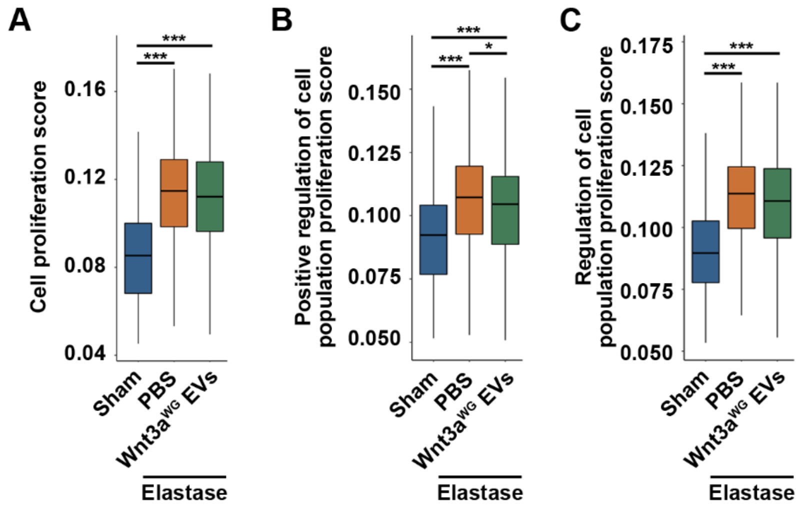

Figure S18. The status of AT1 cell proliferation. Box plots showing the cell proliferation (A), positive regulation of cell population proliferation (B), and regulation of cell population proliferation (C) scores among groups via using gene sets derived from Gene Set Enrichment Analysis database. For box plot, the center line indicates the median; the box limits the upper and lower quartiles; the whiskers represent 1.5 × IQR. IQR, interquartile range. *, $p < 0.05$; ***, $p < 0.001$.

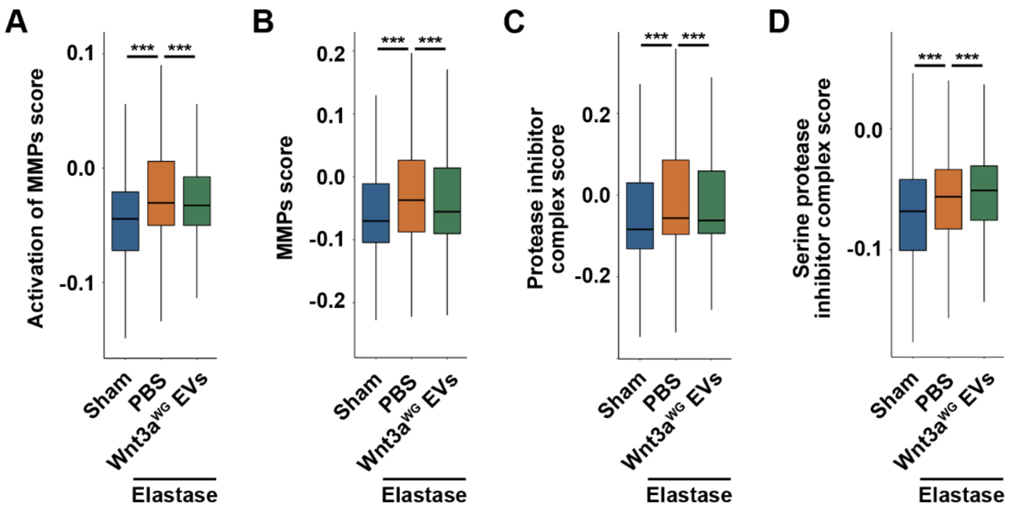

Figure S19. The status of protease and antiprotease systems. Box plots showing the activation of matrix metalloproteinases (MMPs) (A), MMPs (B), protease inhibitor complex (C), and serine protease inhibitor complex (D) scores among groups via using gene sets derived from Gene Set Enrichment Analysis database. For box plot, the center line indicates the median; the box limits the upper and lower quartiles; the whiskers represent 1.5 × IQR. IQR, interquartile range. ***, $p < 0.001$.

Table S1. Primers for qRT-PCR.

Gene	Forward	Reverse
BRACHYURY	TATGAGCCTCGAATCCACATAGT	CCTCGTTCTGATAAGCAGTCAC
POU5F1	CTTGAATCCCGAATGGAAAGGG	GTGTATATCCCAGGGTGATCCTC
AXIN2	AGAAATGCATCGCAGTGTGAAG	GGTGGGTTCTCGGGAAATG
TCF4	GCGCGGGATAACTTGGAAAG	GGATTTAGGAAACTTCGCTGTGT
LGR5	CTCCCAGGTCTGGTGTGTTG	GAGGTCTAGGTAGGAGGTGAAG
ACTB	AGTGTGACGTGGACATCCGCAA	ATCCACATCTGCTGGAAGGTGGAC
Axin2	AGCAGAGGGACAGGAACCA	CACTTGCCAGTTTCTTTGGCT
Tcf4	GTGGGAACTGCCCCGTT	GTTCTAAGAGCACAGGGCAGTTG
Nkd1	TGTTCTCATCCACGCAATGG	GAGCCCCACTCAGGTTCCA
Lgr5	GCGTTCACGGGCCTTCACAG	GGCATCTAGGCGCAGGGATTGA
Gapdh	AGGTCGGTGTGAACGGATTTG	GGGGTCGTTGATGGCAACA
